# Microseek: A Protein-Based Metagenomic Pipeline for Virus Diagnostic and Discovery

**DOI:** 10.3390/v14091990

**Published:** 2022-09-08

**Authors:** Philippe Pérot, Thomas Bigot, Sarah Temmam, Béatrice Regnault, Marc Eloit

**Affiliations:** 1 Institut Pasteur, Université Paris Cité, Pathogen Discovery Laboratory, F-75015 Paris, France; 2 Institut Pasteur, Université Paris Cité, Bioinformatics and Biostatistics Hub, F-75015 Paris, France; 3 Ecole Nationale Vétérinaire d’Alfort, F-94700 Maisons-Alfort, France

**Keywords:** metagenomics, virus, discovery, diagnostic, bioinformatics, pipeline

## Abstract

We present Microseek, a pipeline for virus identification and discovery based on RVDB-prot, a comprehensive, curated and regularly updated database of viral proteins. Microseek analyzes metagenomic Next Generation Sequencing (mNGS) raw data by performing quality steps, de novo assembly, and by scoring the Lowest Common Ancestor (LCA) from translated reads and contigs. Microseek runs on a local computer. The outcome of the pipeline is displayed through a user-friendly and dynamic graphical interface. Based on two representative mNGS datasets derived from human tissue and plasma specimens, we illustrate how Microseek works, and we report its performances. In silico spikes of known viral sequences, but also spikes of fake *Neopneumovirus* viral sequences generated with variable evolutionary distances from known members of the Pneumoviridae family, were used. Results were compared to Chan Zuckerberg ID (CZ ID), a reference cloud-based mNGS pipeline. We show that Microseek reliably identifies known viral sequences and performs well for the detection of distant pseudoviral sequences, especially in complex samples such as in human plasma, while minimizing non-relevant hits.

## 1. Introduction

The development of Next Generation Sequencing (NGS) promoted agnostic identification of known microorganisms and discovery of unknown microbes, in particular viruses. Two important applications coexist and overlap: clinical diagnostic as well as discovering new viruses. The first one aims at identifying known (or closely related) viruses in both human and animals. This approach is generally stringent in order to discard non-viral sequences and viral sequences of environmental origin with poor clinical relevance and to accurately assign a taxonomy to the identified virus [1]. These pipelines must be validated [2] according to national or international regulations in force, and various guidelines are now available [3,4]. The second application, virus discovery, crucially matters to basic research. Indeed, it is important to identify new viruses that may be very different from known ones, for example in arthropod vectors or in wild animal species, notably to anticipate risks of emergence. Discovery of novel viruses is also critical for viral safety issues of commercialized biological products, in which broad range identification of viruses that could be introduced from human or animal raw materials is essential [5,6,7].

In the past years, many dedicated platforms and software were developed. Some of them run on dedicated computational infrastructures [8,9,10] while others directly operate in the cloud, an option which is not always compatible with the confidentiality of medical and industrial data [11,12]. Furthermore, in such bioinformatics tools, databases are often generalist, may include incomplete genomes and may be of lesser stringency [13]. In this context, full exploration of sequence data relies on developing flexible search tools that may bridge existing gaps among available solutions.

Here we present Microseek, a software that is primarily oriented to discovering new viruses while efficiently detecting already known ones. Our approach is grounded into the following observations: (i) most of the genome sequence of viruses encodes proteins, (ii) several protein domains with specific biological functions are conserved (e.g., the viral polymerases) as opposed to the degenerated nature of the genetic code, (iii) it is therefore easier to detect evolutionarily distant viruses based on protein similarity. Further, our pipeline relies on the detection of viral protein genes and takes advantage of the exhaustive and curated viral sequence database RVDB-prot [14], itself derived from the nucleic Reference Virus DataBase (RVDB) [13].

Instead of randomly picking one best match, Microseek taxonomic assignment is based on the Lowest Common Ancestor (LCA), providing a more accurate overview of a sample content. When the sequences share a low identity with sequences in the database, the LCA procedure of Microseek relates the sequence to a higher taxonomic group. Such a case is highly suggestive that the sequence may belong to an unknown organism.

We evaluated and compared the performances of Microseek to Chan Zuckerberg ID (CZ ID, formerly IDseq), a cloud-based metagenomics platform that was recently compared to many other software [12]. We used two datasets representative of common clinical metagenomic matrices called Tissues and Plasma. These two datasets were spiked in silico with three concentrations of sequences from six known human viruses representative of the Baltimore classification (ssDNA, dsDNA, ssRNA+, ssRNA−, dsRNA, and retrovirus). The ability of Microseek to detect new or distant viruses was challenged by spiking pseudo-viral sequences derived from an in silico viral evolution procedure, generating three so-called Neopneumovirus sequences with three distinct evolutionary distances within the Pneumoviridae family.

## 2. Materials and Methods

### 2.1. Pipeline

#### 2.1.1. Input Data and Cleaning

The pipeline uses as input short reads raw fastq files, single or paired end, which can be compressed in several common formats. Fastq are trimmed using AlienTrimmer 0.4.0 (accessed date: 28 July 2022) [15] with default options -k 10 -m 5 -l 50 -p 80 -c 012 -q 20 (5 mismatches allowed, minimum length of 50, quality cutoff of 20, 80% of the read above this cutoff). These default options can be changed in the settings, according to user’s needs.

#### 2.1.2. Reads Normalization, Assembly and Translation

Read coverage is then normalized by down-sampling reads localized in deeply sequenced loci, using BBnorm 36.11 [16] with options target = 100 min = 1 (target average depth of 100×, minimum depth of 1). This stage of “digital normalization”, when quantitative information is secondary [17], has been shown to be a major factor in increasing performance and lowering memory consumption and allows the assembly of longer contigs.

The resulting remaining reads are then assembled using Megahit 1.1.2 (accessed date: 28 July 2022) [18] with options—min-contig-len 100 (keeping only contigs with a length of 100 nucleotides). Reads are mapped back to contigs, and those unassociated with contigs are kept as “singletons”. This mapping is performed using Bowtie 2.1.0 (accessed date: 28 July 2022) [19] using—fast option.

Both contigs and singletons are then translated into proteins by an ad-hoc program that looks for ORFs among the six reading frames, keeping sequences with a length greater than 15 amino acids [20]. At this step, original nucleic sequences associated with protein sequences are kept.

#### 2.1.3. Taxonomic Assignation of Protein Sequences

Microseek uses three levels of blast homology searches (called B1, B2, and B3) based on three different databases. This sequential pattern of queries uses relaxed parameters in B1 on a viral restricted database in order to detect distant sequences, then invalidates false positives in B2 and B3 on more generalist databases. For protein searches, we used Diamond blastp [21] instead of NCBI blastp tool, as it approximates a blastp with a very low loss of sensitivity while increasing the query speed up to 40,000 times and keeping the same results format and metrics as NCBI blastp.

B1: The protein sequences predicted at the previous step are queried using Diamond blastp 0.9.26 against the latest release of RVDB-prot (currently 23.0) [14], with a maximum e-value requirement of 5. Tabular output is annotated, subject sequences (i.e., potential matches) are associated with a given taxon using taxadb. Results are then combined, generating LCA annotation (corresponding to the common ancestor of all the best matches). A script generates sub-sequences that will be used as queries during the next stage: they correspond to the portion that aligned with a match in the database, commonly known as High-scoring Segment Pair (HSP).

B2 is the first validation step: NCBI NR protein databank is queried with Diamond, with a maximum e-value of 100 (this value can be defined by user) using the sub-sequences produced during B1 as queries, and further annotated with LCA. Sequences having a LCA that does not belong to Viruses (taxid 10,239) are discarded, and hits corresponding to “artificial sequences” (taxid 81,077) are not considered for the inference of the LCA (else, these sequences would not be considered as viral, as artificial sequence is not inside “Viruses” taxon). For the remaining sequences, the original nucleic portion of the sequence (corresponding to the ORF aligned to the first B1 match) is then retrieved for the final validation step.

During B3, a blastn (Blast+ 2.6.0, parameter task = blastn, no criterion applied on e-value) is finally performed using the nucleic sequences produced in B2 to query NCBI NT databank. Only sequences with a viral hit (taxid 10,239) are conserved. This step allows to eliminate sequences that have a better assignation with non-coding sequences and allows, for example, to filter out cellular sequences present in certain viral genomes like in pestiviruses [22]. These three successive assignment levels (B1, B2 and B3) contribute to the elimination of inherent false positives, without replacing human expertise of the final results.

To try to broaden the range of detection of viruses to very distant viruses, ORFs with no match in B1, B2 and B3 and whose size is greater than a user-defined threshold (default setting: 666 amino acids, i.e., 2000 nucleotides) are tested against the RVDB-prot-hmm database using HMMER (with—domtblout parameter). The resulting HMM H4 file is a list of homologous clusters with associated keywords and LCA retrieved from RVDB-prot-hmm annotation database [14], along with the best probability scores (best_proba) and the size of the longest matching domain (greater_length).

#### 2.1.4. Output

Two raw tables are outputted. They contain, respectively, results of steps B2 and B3, providing several information: original sequence name, original nucleic sequence, translated sequence; and information about B1 and B2 results: e-value, LCA, percentage of amino-acid identity, difference of taxonomic level assignation between B1 and B2, length of queries that matched with sequences contained in the database (query coverage), and protein types. In addition, the presence of an ORF encompassing the whole sequence length is reported, as it can help to differentiate exogenous viruses from endogenous viral sequences. Indeed, viral sequences integrated in host genomes are not constrained by selection pressures and often contain stop codons. The information of ORFs that cover the whole sequence is, therefore, a good indicator that the identified virus sequence does not correspond to an integrated viral sequence, which are frequent in arthropod genomes for example. The HMM output is presented separately in a H4 raw table.

A dynamic graphical webpage is finally generated: it contains a HTML web page that allows the exploration of all validated results with the information present in table B3, with several useful features to select, sort, visualize the taxonomic distribution (via a Krona representation [23]), or export relevant sequences. This output is very useful and was conceived to facilitate the exploration of the data by non-bioinformaticians.

### 2.2. Matrix Datasets

Two datasets from pre-existing clinical metagenomic experiments, reflecting two different types of matrices, were used. The first one, called Tissues, was derived from transcriptomic data generated from human biopsies, from which sufficient nucleic acids input can be extracted to construct NGS libraries, which reduces the need for amplification procedures and subsequent co-amplification of a background of unspiked sequences from laboratory components [24]. This dataset was composed of a random selection and mixing of 50 million reads generated with the SMARTer Stranded Total RNA-Seq Kit v2—Pico Input Mammalian (Takara) from the transcriptome analysis of 4 human biopsies (kidney, liver, nymph node and brain) in which no known human pathogen was found.

The second dataset, called Plasma, reproduced a virome analysis from human plasma, with low nucleic acids yields after extraction of nucleic acids after hydrolysis of non-encapsidated nucleic acids, which needed random pre-amplification before library construction. In addition, phages populations plus other probable translocation of viral sequences in blood made the plasma to have a much lower host vs. non-host sequences ratio leading to an over-amplification and detection of a background made of unspiked sequences. This second dataset consisted of a random selection and mixing of 50 million NGS reads generated from two human plasma in which no known human pathogen was found and was generated from total nucleic acids (DNA and RNA) with the NEBNext Ultra II DNA Library Prep Kit (NEB) after a reverse transcription and pre-amplification phases using MALBAC [25]. For both datasets, only reads of good quality after trimming were kept. Names of the original reads were modified, and reads were mixed-up to guarantee personal data protection.

### 2.3. Selection and In-Silico Spike of Viral Sequences

Six known human viruses representing six groups of the Baltimore classification were selected as input reference sequences for spiking experiments. ssDNA: Human parvovirus B19 (NC_000883.2); dsDNA: Human gammaherpesvirus 4 (known as Epstein Barr Virus (EBV); V01555.2); ssRNA+: Enterovirus B (Coxsackievirus B6; AF039205.1); ssRNA-: Human respiratory syncytial virus A (HRSV-A; JF920069.1); dsRNA: Mammalian orthoreovirus type 1 (10 segments: X61586.1, M18389.1, M24734.1, M10260.1, AF174382.1, AF129820.1, AF461682.1, AF490617.1, L19774.1, AF378003.1); and retrovirus: Human immunodeficiency virus type 1 (HIV-1; NC_001802.1).

Two virus template files were defined from the list of viruses, namely “Genome template” and “Transcripts template” and used as starting material for generating reads to be spiked. The following principle was followed in the Tissues dataset, which mimicked a transcriptome approach (RNAseq), both genomic and messenger viral RNAs sequences served as template in the proportion 90% and 10%, respectively, to reflect the content of active replicating viruses. For the Plasma dataset, which mimicked a virome approach where viral DNA and RNA protected by capsids are sequenced, only the genomic sequences served as a matrix, reflecting the content of viral particles.

From each viral sequence, spiked fastq reads were generated using ART version MountRainier 2016-06-05 (accessed date: 28 July 2022) [26]. ART produces in silico sequencing reads that respect pre-determined error profiles, which could either be built-ins or created from a user-provided fastq file (with the tool art-profiler-illumina, provided by the ART package). The latter option was used to generate two error profiles, respectively, for the Tissues and Plasma datasets, according to the quality of the reads of each dataset. Then, art-profiler-Illumina was used to produce the defined number of reads following the rules defined below. Generated reads were finally spiked in the corresponding datasets, at a random position of the fastq file.

Three in silico viral spiking loads, namely dilution 1 (d1), dilution 10 (d10) and dilution 100 (d100) were set-up. The number of spiked sequences at d1 was calibrated to mimic clinical viral loads of 10^5^ copies/100 mg of tissue [27] and of 10^2^–10^3^ copies/mL of plasma as previously assessed with reference spike viruses [28]. Detailed rules and proportion of spiked sequences are presented in the Supplementary Table S1.

### 2.4. Creation of Distant Viral Sequences

Four species of *Orthopneumovirus* were used as a starting group to derive distant viral sequences: Murine orthopneumovirus (AY729016.1), Bovine orthopneumovirus (AF092942.1), Human orthopneumovirus A (HRSV-A2, KT992094.1), and Human orthopneumovirus B (HRSV-B1, AF013254.1). Corresponding protein sequences of the six main proteins (Nucleoprotein N, Phosphoprotein P, Matrix M, Glycoprotein G, Fusion F, and Polymerase L) were aligned with MAFFT 7.467 using—auto mode; and phylogeny was reconstructed with IQ-TREE 2.0.6 (accessed date: 28 July 2021) with options -m JTT -asr (evolutionary model Jones-Taylor-Thornton, ancestral sequence reconstruction). The Newick phylogenetic tree file was text-edited to branch the three new viruses, so called Neopneumovirus-1, Neopneumovirus-2 and Neopneumovirus-3.

This tree, and the ancestral sequence previously inferred, were used as input of Seq-Gen 1.3.4, with options -m JTT -k1 (JTT model, and ancestral sequence setting) to produce synthetic sequences. The in silico evolution method generates random mutations conforming to the evolutionary model throughout the sequence. Protein sequences were then back translated to nucleic sequences using random codons, and the resulting nucleic sequences were concatenated, keeping the same gene order than in real pneumoviruses, to obtain pseudo-genomes. The range of protein (AA) and nucleic (NT) homologies of Neopneumovirus-1, -2 and -3 for the N, P, M, G, F, and L genes, compared to the Human respiratory syncytial virus A, were as follows: Neopneumovirus-1: 36.6–70.5% (AA), 44.8–63.8% (NT); Neopneumovirus-2: 27.3–56.2% (AA); 41.7–57.8% (NT); Neopneumovirus-3: 21.8–44.5% (AA); 38.5–54.2% (NT) (Supplementary Table S2).

### 2.5. Chan Zuckerberg ID (CZ ID)

Data analysis with CZ ID was conducted online using pipeline v7.0 (accessed date: 4 April 2022) [12] with default parameters that include human genome filtering. Output files have been filtered to keep only eukaryotic viruses.

### 2.6. Filtering Pre-Existing Content and Mapping

Outputs from Microseek and CZ ID were analyzed after filtering-out the sequences that correspond to taxonomies previously found in the unspiked dataset. Reads alignment onto reference genomes was done with the Geneious Prime 2022.1.1 mapper (accessed date: 22 June 2022) using High Sensitivity parameters.

### 2.7. Data and Software Availability

The following material is available online at Zenodo (https://doi.org/10.5281/zenodo.6937000 (accessed on 28 July 2022); Creative Commons Attribution 4.0 International License). This record provides seven archives: raw fastq files corresponding to the unspiked (2 datasets); spiked with known viruses (6 datasets); spiked with Neopneumoviruses (12 datasets); additional tick sample used in discussion and negative control experiments; Neopneumovirus sequences (fasta files) and all Microseek outputs (40 folders).

## 3. Results

### 3.1. Overview of the Pipeline Architecture

**Figure 1:**
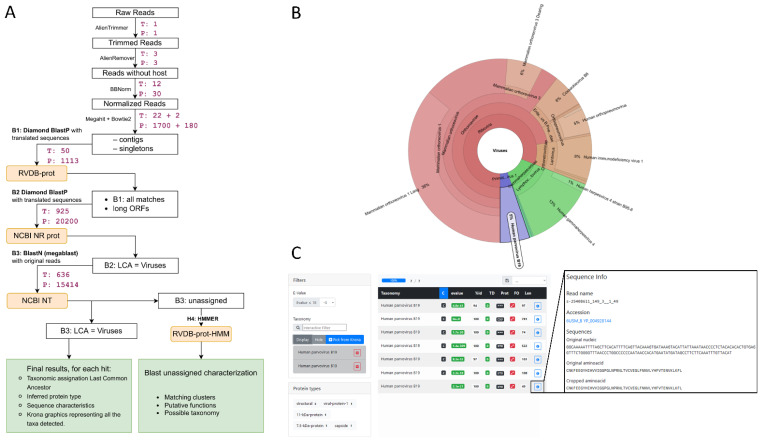
Microseek pipeline steps and output examples. (**A**) Step details of the pipeline. For each operation, software and database names are indicated. The times on the right represent the cumulated duration of each step (in minutes), respectively, for the Tissues (T) and Plasma (P) datasets and corresponding to spiked experiments at d1. For instance, for Plasma dataset, B1 step would take 1113 min if it was run with a single CPU as a unique chunk. (**B**) Example of a result browser webpage. The Parvovirus B19 taxon was selected from the Krona chart as an example. (**C**) Resulting list of hits of the Tissues dataset after filtering on Human Parvovirus B19 with e-value ≤ 10^−3^, showing information such are the existence of contigs (C), e-value, %ID, length of the sequence and protein names. Information boxes associated to each hit give the nt and aa sequences and allow direct access to the corresponding NCBI entries.

We present Microseek, a pipeline with a graphical interface for virus identification and discovery to be run with Snakemake [29] (Figure 1). It takes advantage of a well-defined, curated and regularly updated reference database made of protein viral sequences (RVDB-prot [14]). As so, after common QC and first-step procedures consisting mainly in normalization and sequences assembly into contigs, the first specific step of the pipeline is the translation of the nucleotides sequences into coding amino-acid sequences and the search for similarity within the RVDB-prot database (Figure 1A, step B1). Hits are then filtered applying user-defined e-value criteria and the specificity of the remaining sequences is validated against the entire NCBI NR database (Figure 1A, step B2). A final blastn procedure on the NCBI NT database (Figure 1A, step B3) mainly aims to exclude host sequences recombined in viral genomes, which would have presented alignment homologies with viral sequences in the previous steps. Microseek consider all co-best hits, summarized by their LCA. At the end, a visual representation based on Krona is given (Figure 1B,D), together with a home-made HTML interface which allows for easy sequences selection, filtering and retrieving directly from Krona, along with public annotations (taxonomy, related accession numbers, protein names) provided (Figure 1C,E). All the buttons are clickable, either for data sorting or selection. For example, all sequences of a given taxonomy can be selected, or all sequences of a given protein type, or any combination can be sorted according to their size or identity percentage. Taxonomy names and accession numbers are also clickable, allowing for fast retrieval of all taxonomic information or sequence entry data (see output_microseek.tar.xz file at https://doi.org/10.5281/zenodo.6937000, accessed on 28 July 2022).

In its structure, Microseek consists in a pipeline specification, several scripts written in Python 3.6.9 (accessed date: 28 July 2022), and a provided Singularity container including all the needed dependency softwares. It requires a machine running Linux, x86_64 platform, and preferably runs on a cluster. User indicates in a yaml (formatted plain text) configuration file some parameters about the sample: path and name of the fastq files, sequencing type (paired or single ends), and name of the experiment. Snakemake manages the clusters options. Running the pipeline requires public databases (NCBI NR, NCBI NT, rvdb-prot, taxadb), as well as the protein types database generated from RVDB-prot (script for automatic generation is provided) in SQLite format. The Figure 1A illustrates the time/CPU required for the exemplified analysis (spiked Tissues d1 and spiked Plasma d1).

### 3.2. Characterization of Pre-Existing Unspiked Datasets and Negative Control

To define the pre-existing content of viral sequences in real clinical matrices, the two datasets were analyzed with Microseek and CZ ID in the absence of additional in silico viral spike. In the unspiked pre-existing Tissues dataset, only one read wrongly attributed to Hepatitis C virus was reported by Microseek because of a wrong reference sequence in NCBI, while no background was visible in CZ ID output. By contrast, in the unspiked pre-existing Plasma dataset, a broad-spectrum of virus-related sequences was observed by both methods, including non-pathogenic viruses carried by human plasma (mostly bacteriophages) and viral nucleic acids introduced by reagents and contaminating the NGS libraries (Supplementary Table S3). To describe and quantify the level of contaminating viral sequences carried by reagents, a negative control (water) was also sequenced (Supplementary Table S3).

### 3.3. Detection of Known Viral Sequences

To evaluate the performances of Microseek in detecting known viral sequences, given amounts of viral reads were spiked in the datasets. The horizontal coverage of spiked reads along the genome of the six reference viruses ranged from 7.5–99.8% (dilution 1; d1), 2.0–56.9% (dilution 10; d10) and 0.3–16.1% (dilution 100; d100) for the Tissues experiments, and from 51.1–99.8% (d1), 6.9–78.7% (d10), 1.0–20.4% (d100) for the Plasma experiments (Table 1). We deliberately chose not to fully cover each virus with spiked sequences in order to better reflect real life metagenomic results.

The 50 M reads datasets containing the spiked sequences were analyzed with Microseek and CZ ID, and the results obtained were compiled (Table 1). Almost all of the 6 viruses were detected by both Microseek and CZ ID at the species level in the Tissues and Plasma datasets at dilutions d1, d10 and d100. Only the Parvovirus B19 was missed at the lowest d100 concentration in Plasma with CZ ID. HIV-1 detection by Microseek was lower than HIV-1 detection by CZ ID in all experimental conditions. HIV-1 was identified at the genus level by Microseek in plasma at d100 while CZ ID identified correctly the species. Single sequences of primate *Herpesviridae* hits attributed to Macacine gammaherpesvirus, Gorilla lymphocryptovirus 1 or Pan troglodytes lymphocryptovirus 1 (Family: Herpesviridae, Genus: *Lymphocryptovirus*), Macacine betaherpesvirus 8 (Family: Herpesviridae, Genus: *Cytomegalovirus*) or Macacine gammaherpesvirus 11 (Family: Herpesviridae, Genus: *Rhadinovirus*) were detected by both Microseek and CZ ID but did not compromise the correct identification of human EBV at the species level given the high recovery rate of EBV-specific sequences.

**Figure 2:**
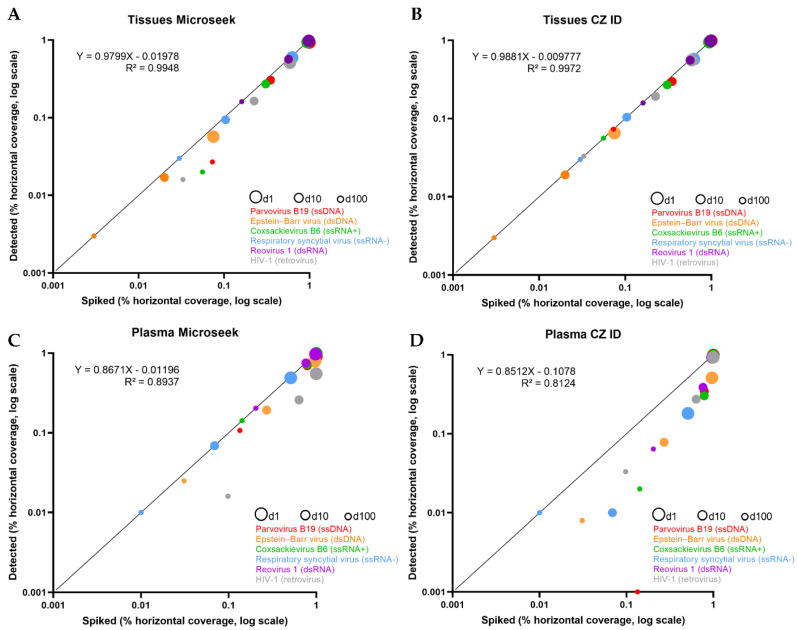
Detection of six known viruses (spiked vs. detected). Scatter plots of spiked (*X* axis) vs. detected (*Y* axis) percentage horizontal coverage for the 6 known viruses of the study, for the Tissues (**A**,**B**) and Plasma (**C**,**D**) experiments. Each virus is depicted by 3 points, corresponding to the d1 (large circles), d10 (intermediate circles) and d100 (small circles) experiments, and is associated with a specific color (red: Parvovirus B19; orange: Epstein-Barr virus; green: Coxsackievirus B6; blue: HRSV; purple: Reovirus-1; grey: HIV-1). The identity line (Y = X) is represented in black.

The horizontal coverages of spiked vs. detected sequences were compared (Table 1 and Figure 2). In the Tissues experiments in which all viruses were detected, almost all spiked sequences were recovered by Microseek and CZ ID, whatever the dilution (Table 1). Accordingly, slopes of the linear regressions were close to 1 (Microseek: 0.979; CZ ID: 0.988) with intercept values close to 0 (Microseek: 0.019; CZ ID: 0.009) (Figure 2). By contrast, recovery of spiked sequences was generally lower in the Plasma experiments. Microseek was able to detect all viruses while CZ ID missed the Parvovirus B19 at d100 (Table 1). Although the slopes of the regressions were similar (Microseek: 0.867; CZ ID: 0.852), the intercept value was much closer to zero with Microseek (0.011) than with CZ ID (0.107), indicating a better overall detection by Microseek in the Plasma experiments (Figure 2).

The specificity of detection, which is reflected by the list of non-spiked but detected sequences, was also documented. In the Tissues experiments, only three non-specific Macacine Herpesviridae sequences were detected by Microseek at dilution 1 while one Macacine Herpesviridae sequence at d1 and one Hylobates *lymphocryptovirus* sequence at d10 were detected by CZ ID. In Plasma, a more extensive list of residual hits was recorded for the two pipelines. The main noisy signals were attributed to phages (*Caudovirales* including Siphoviridae, and Microviridae), environmental or unclassified viral isolates, plus other poorly characterized RNA or DNA viruses (Supplementary Table S4). The number of non-spiked but detected taxa (whatever their taxonomic level), which is a measure of the diversity of the background reported in the outputs, was lower with Microseek (ranging between 6 and 9 taxa) that with CZ ID (157 to 161 taxa) (Supplementary Table S4).

### 3.4. Detection of Novel, Distant Viral Sequences

**Figure 3:**
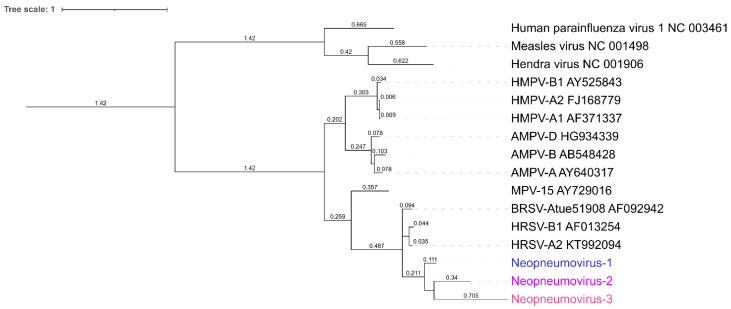
Phylogeny of Neopneumoviruses. Protein sequences of the polymerases were aligned with MAFFT v7.450, and the phylogeny was reconstructed with IQtree 2.0.6 with model of substitution JTT + F + G4. The resulting tree was rooted using members of the Paramyxoviridae family as outgroup. AMPV: Avian metapneumovirus; HMPV: Human metapneumovirus; MPV: Murine orthopneumovirus; BRSV: Bovine orthopneumovirus; HSRV: Human respiratory syncytial virus. Accession numbers are indicated in the figure. Numbers on the branches represent their lengths.

**Figure 4:**
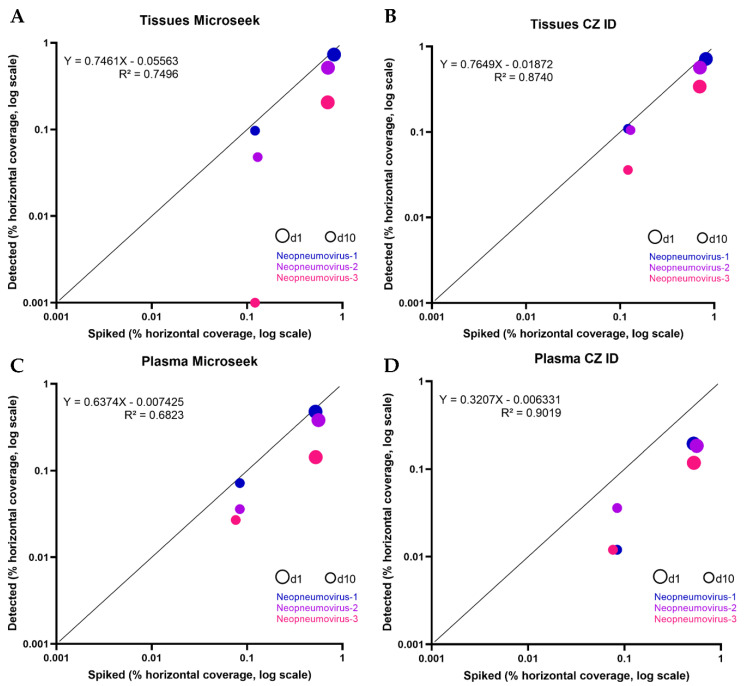
Detection of three Neopneumoviruses (spiked vs. detected). Scatter plots of spiked (*X* axis) vs. detected (*Y* axis) percentage horizontal coverage for the 3 fake Neopneumoviruses of the study, for the Tissues (**A**,**B**) and Plasma (**C**,**D**) experiments. Each virus is depicted by 2 points, corresponding to the d1 (large circles) and d10 (intermediate circles) experiments, and is associated with a specific color (blue: Neopneumovirus-1; purple: Neopneumovirus-2; pink: Neopneumovirus-3). The identity line (Y = X) is represented in black.

To assess the ability of Microseek to detect distant viruses, we simulated the evolution of three new viruses, so-called Neopneumovirus-1, -2 and -3, derived from the genus *Orthopneumovirus* (Figure 3 and Supplementary Table S2). Neopneumovirus reads were spiked to a number corresponding to dilution 1 (107 reads for Tissues, 67 reads for Plasma) and to dilution 10 (11 reads for Tissues, 7 reads for Plasma), following the same random generation process as done for known viruses (d1: reference horizontal coverages of 70.1–81.6% in Tissues and of 52.2–56.1% in Plasma; d10: reference horizontal coverages of 12.1–12.9% in Tissues and of 7.6–8.4% in Plasma). The 12 corresponding spiked datasets were analyzed with Microseek and CZ ID (Table 2 and Figure 4). Dilution 100 was not tested because the limit of detection of Neopneumoviruses was reached at d10.

In the Tissues experiments, part of the sequences from all three Neopneumoviruses were detected with both Microseek and CZ ID (Table 2). At d1, horizontal coverages obtained with Microseek was better for Neopneumovirus-1 (73.3% vs. 71.5%), while CZ ID gave better detections of Neopneumovirus-2 (51.6% vs. 56.6%) and Neopneumovirus-3 (20.6% vs. 34.0% for Microseek and CZ ID, respectively). Microseek made taxonomic assignments at the family (Neopneumovirus-1 and -2) or at the genus (Neopneumovirus-3) levels, whereas taxonomic assignations with CZ ID were made at the family level only. At d10, Microseek detected Neopneumovirus-1 and -2 at the family and genus levels, while CZ ID did all identifications at the family level and Neopneumovirus-3 was missed by Microseek (Table 2). Overall, the horizontal coverages of spiked vs. detected sequences in the Tissues experiments were slightly higher with CZ ID that with Microseek, as indicated by the slopes (Microseek: 0.746; CZ ID: 0.764) and the intercept values (Microseek: 0.055; CZ ID: 0.018) (Figure 4). No unspiked sequence was recorded.

In the Plasma experiments, all three Neopneumoviruses were detected by Microseek and CZ ID at d1 and d10, but horizontal coverages were higher with Microseek at d1 (47.7% vs. 19.4% for Neopneumovirus-1; 38.1% vs. 18.4% for Neopneumovirus-2; 14.3% vs. 11.8% for Neopneumovirus-3) and higher or equivalent to CZ ID at d10 (7.2% vs. 1.2% for Neopneumovirus-1; 3.6% vs. 3.6% for Neopneumovirus-2; 2.7% vs. 1.2% for Neopneumovirus-3) (Table 2). Taxonomic assignments with Microseek were done at the species (Neopneumovirus-3 at d1 and Neopneumovirus-2 at d10), genus (Neopneumovirus-1 and -3 at d10) and family (Neopneumovirus-1 at d1 and Neopneumovirus-2 at d1) levels, when CZ ID identifications were made at the genus level at d1 and at the species (Neopneumovirus-1) and family (Neopneumovirus-2 and -3) levels at d10 (Table 2). Overall, the horizontal coverages of spiked vs. detected sequences in the Plasma experiments were better with Microseek that with CZ ID, with slopes much closer to 1 (Microseek: 0.637; CZ ID: 0.320) and similar intercept values (Microseek: 0.007; CZ ID: 0.006).

A list of unspiked hits of similar composition to that observed in the spiking of known sequences was recorded (Supplementary Table S5), and again, its diversity was significantly lower with Microseek (range: 5–9 taxa) than with CZ ID (range: 150–170 taxa).

### 3.5. Signal-to-Noise Ratios in Complex Samples

**Figure 5:**
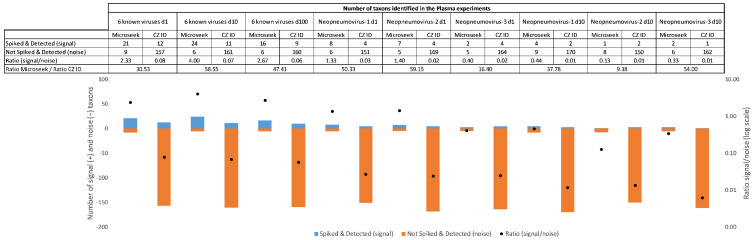
Signal-to-noise ratio in the Plasma experiments. Histograms below the table represent the signal (blue bars with counts above zero on the primary axis) vs. noise (orange bars with counts below zero on the primary axis) and associated signal-to-noise ratios (black dots on the secondary axis).

In challenging experimental conditions such as Plasma, the signal-to-noise ratio of taxons, defined as the ratio of spiked and detected to not spiked but detected taxons, were used as an indicator of the ease of extracting relevant information from a list of primary hits. In the spike-in experiments with known viral sequences, the signal-to-noise were 30 to 58 times more favorable with Microseek than with CZ ID for all dilutions (30.5-fold at d1; 58.5-fold at d10 and 47.4-fold at d100) (Figure 5). Better signal-to-noise ratios with Microseek in comparison to CZ ID, ranging from 9.3-fold (Neopneumovirus-2 at d10) to 59.1-fold (Neopneumovirus-2 at d1), were also observed in the Neopneumoviruses experiments (Figure 5).

### 3.6. Limit of Detection of Distant Viral Sequences in Complex Samples

The distribution of spiked vs. detected reads along the N/P/M/G/F/L genes of Neopneumoviruses in Plasma was used to document the maximum evolutionary distance compatible with detection (Supplementary Figure S1 and Supplementary Table S2). At d1, Neopneumovirus-1 was detected through the N/P/M/G/F/L genes with Microseek and the N/G/F/L genes with CZ ID, with spiked reads known to cover all six genes. Detection of Neopneumovirus-2 was done through to the N/P/G/F/L genes with Microseek, compared to only the N/G/L genes with CZ ID although spiked reads targeted all genes. Neopneumovirus-3 was detected through to the M/L genes with Microseek while CZ ID was able to see the N/M/F/L genes. At d10, Neopneumovirus-1 G/F/L spiked reads were detected with Microseek when CZ ID detected only the F genes. Neopneumovirus-2 F/L genes were detected with both pipelines, while Neopneumovirus-3 detection was done through the L gene only with Microseek and the N gene only with CZ ID. Overall, the most distant proteins detected by Microseek in challenging conditions were the L protein of Neopneumovirus-3 at d10 (42.2% AA Id with the L glycoprotein gene of HSRV) and the G protein of Neopneumovirus-2 at d1 (27.3% AA Id with the G glycoprotein gene of HSRV). Under the same experimental conditions, CZ ID did not detect the L protein and detected fewer sequences from the G protein (Supplementary Figure S1 and Supplementary Table S2).

### 3.7. HMM Profiles

Output data of the HMM branch of Microseek is presented in the Supplementary Table S6. No hit was recorded for the Tissue experiments. In Plasma, the same set of three HMM clusters FAM001703 (lectin-related binding domain), FAM001286 (polymerase) and FAM021277 (ribonuclease) was observed in all conditions and was therefore interpreted as non-informative background.

## 4. Discussion

In this work, we introduced Microseek, a Snakemake-based pipeline for virus identification and discovery taking advantage of a reference viral protein database, RVDB-prot [14]. The performances of Microseek were illustrated with clinical metagenomics datasets in which reads from known and new viruses were spiked at known concentrations. This experimental procedure has the advantage of testing realistic situations, while controlling parameters of interest such as the number of reads and genome coverage. The number of spiked sequences in the Tissues and Plasma datasets was calibrated to mimic actual infection as tested routinely in the laboratory. CZ ID, an open-source cloud-based pipeline developed by the Chan Zuckerberg Initiative [12] was used for comparison’s sake. CZ ID has previously demonstrated very good performances compared to 22 analysis tools [12] and can therefore be considered as a best-in class in the field.

The detection of known viral sequences was achieved with Microseek with an overall good sensitivity on a wide range of spike concentrations despite, however, a loss of sensitivity for the detection of HIV-1. In complex matrices with a high background of unspiked sequences such as plasma, as low as one read spiked in a 50 million reads dataset was detected for HRSV. Microseek succeeded in detecting the Parvovirus B19 starting from 5 spiked sequences, where CZ ID did not in limit dilution represented by the d100 condition, which reflects low viral loads found in some clinical situations. Overall, the detection performances of spiked sequences in Plasma were better with Microseek (slope: 0.867; intercept: 0.011) than with CZ ID (slope: 0.852; intercept: 0.107). This discrepancy may come from the random subsampling of 1 million sequences made by CZ ID among non-human sequences, which appears to be a handicap of CZ ID for samples made of a large fraction of non-human sequences even when viral loads are compatible with qPCR detection (10^2^–10^3^ copies/mL). Conversely, samples in which the fraction of non-human reads was minimal, such as human tissues, benefited well from the filtering of human sequences implemented in CZ ID (R^2^ = 0.997) and in Microseek (R^2^ = 0.994). In this respect, the case of HIV-1 illustrates the impact of endogenous viral sequences within the human genome, which can lead to the unwanted removal of exogenous viral sequences, especially lentivirus. The relevance of host filtering and subsampling steps, often presented as an effective way to reduce the computing demand, should therefore be carefully considered.

It is noteworthy that many wild animal and arthropod genomes have not yet been sequenced or are only roughly annotated. In an attempt to get rid of the effect of human sequences depletion, we analyzed a *Rhipicephalus* tick sample known to be infected with the Cataloi Tick Quaranjavirus (CTQV, Family: Orthomyxoviridae, Genus: *Quaranjavirus*) [30]. No reference tick genome was used for host filtering. All five viral segments of CTQV (PB1, PB2, PA, HA, and NP) were successfully detected with Microseek with a 100% horizontal coverage. CZ ID was able to detect the PB1 and PA segments with 100% horizontal coverage, the HA and NP segments with 84% and 20.2% horizontal coverage, respectively, but missed the PB2 segment (Supplementary Figure S1). The signal-to-noise ratios of Microseek (3 *Quaranjavirus* taxa vs. 66 other taxa) and CZ ID (6 vs. 86) were not much different. This experiment on non-human samples suggests that Microseek can remain a powerful discovery tool even in the absence of a reference host genome.

Microseek reports the Last Common Ancestor (LCA) instead of arbitrarily providing one of the best hits when several taxonomic assignments with comparable scores are found. For example, if a query has identical matches in the database with several viruses belonging to different families, the LCA will not be precise (for instance «Viruses») but will be more accurate regarding the robustness of the taxonomic assignation than random picking of one species. Conversely, the identification of a sequence at the virus species level would strengthen the taxonomic assignation of the species. Reporting the LCA thus prevents reporting false assignments and opens the way to characterizing novel viruses.

An important feature of Microseek is its protein-based search, assuming viral genomes are compact and contain mainly coding sequences. If needed, after primary identification, the identification of non-coding sequences can be achieved by re-analyzing the raw nucleotide data to identify sequences that do not code for proteins (regulatory sequences, packaging signals, intergenic sequences, untranslated RNA transcripts…). Indeed, due to the conservation of protein functions across biological organisms, research based on protein analyzes present the advantage of being able to detect distant viruses presenting with low nucleotide homology. By delivering results in a more functional way, a protein-based search may also contribute to increase the signal-to noise ratio and help identify distant viruses hidden in large output files. This feature was exemplified in our work, in which the signal-to-noise ratio of spiked vs. not spiked sequences of Neopneumoviruses was minimal with Microseek in comparison to CZ ID in a complex matrix such as plasma.

The ability of Microseek to detect distant viral sequences was evaluated by generating and spiking three so-called Neopneumovirus sequences, related to the genus *Orthopneumovirus*. Conceptually, the three evolutionary distances tested were designed to challenge the detection of new viral species within a known genus (Neopneumovirus-1) but also a new genus within a known family (Neopneumovirus-2) or even possibly higher taxonomy levels (Neopneumovirus-3). The detection of Neopneumoviruses appears to depend on the evolutionary distance from the nearest known HRSV sequence, but also on the number of spiked reads. Indeed, among the six N/P/M/G/F/L tested genes, Microseek retained the ability to detect the G and L genes in challenging conditions, whether this challenge came from a lower sequence homology (Neopneumovirus-1 vs. Neopneumovirus-2 at d1) or from a lower number of sequences (Neopneumovirus-1 at d1 vs. Neopneumovirus-1 at d10). The G glycoprotein, which is determinant for the tropism of infection of HRSV, has expectedly the lowest level of conservation among the N/P/M/G/F/L evolutionary-derived genes in our Neopneumovirus experiment (Supplementary Table S2). In general, envelope glycoproteins are known to be more difficult to detect because more variable than conserved polymerase sequences in metagenomics experiments [31]. When the initial number of reads was not limiting, at d1, Microseek succeeded at detecting two sequences from the G glycoprotein of Neopneumovirus-2 (27.3% AA Id with the G glycoprotein gene of HSRV) (Supplementary Figure S1 and Supplementary Table S2). One sequence of the G glycoprotein of Neopneumovirus-1 (36.6% AA Id with the G glycoprotein gene of HSRV) was also detected by Microseek in challenging condition at d10. The subsampling step introduced by CZ ID and discussed above does not allow for a strict comparison with Microseek regarding the sensitivity to the initial number of sequences. However, at d1, the G glycoprotein of Neopneumovirus-2 was detected with CZ ID with only one sequence, and no detection of the G glycoprotein of Neopneumovirus-1 was reported at d10. The detection of distant viral proteins by CZ ID down to the empirical limit of 70% identity previously reported supports the remark of CZ ID’s authors that a protein search could have a broader range of detection [12]. In line with that, the proposed taxonomic assignations from Microseek in the Plasma experiments were, in some cases, one level higher than taxonomic assignations coming from CZ ID (family for Neopneumovirus-1 and -2 with Microseek vs. genus with CZ ID at d1 for example). This supports the idea that, in complex samples such as plasma, Microseek based on LCA can help supporting the presence of new or distant viral sequences. In contrast, it is noteworthy that tools based on k-mer composition (Kraken [32], Centrifuge [33]) or detection of predefined specific markers (Metaphlan 2 [34]) were unable to detect Neopneumoviruses sequences and even some sequences from known viruses (Supplementary Table S7).

Due to evolutionary constraints, the functionally important regions in proteins tend to be relatively conserved [35]. Therefore, protein analysis can allow the use of protein motif libraries such as Hidden Markov Model (HMM) profiles. Each release of RVDB-prot contains a comprehensive HMM database of viral proteins [14]. In our experiments on human samples, the search for HMM patterns from unassigned contigs longer than 2000 nt did not bring any new information compared to the blast approach (Supplementary Table S6). In the *Rhipicephalus* tick sample, a single HMM cluster attributable to *Riboviria* (double stranded RNA-binding domain) was document, which requires further investigation (Supplementary Table S6). Indeed, the end-user can adjust the search parameters to match the nature of the samples and the purpose of his search. For example, if the user expects to find a protein of a certain family or clade, and if this protein is not found by the BLAST-based steps, the HMM search can be used with a very low threshold (sequences with a short minimum length) and thus lead to analyze many output sequences. The result will be a sortable list of all these sequence segments with annotations, including the protein type and associated probability scores. In our experiments, lowering the minimum sequence size from 2000 nt to 500 nt would not have changed the results of the Tissue experiments (because no unassigned sequence was more than 500 nt), but would have required testing up to 17,000 extra sequences in B2 and B3 in the Plasma experiments. Therefore, the setting of the HMM parameters is a trade-off between sensitivity and computing demand. The HMM approach in its current form should be seen as an optional advanced exploratory tool.

Microseek was used during the past years for the diagnosis of a human encephalitis case of unknown origin leading to the identification of Umbre orthobunyavirus, a novel arbovirus for the first time described in humans [27], and for the diagnosis of the first case of lethal encephalitis due to a bat Lyssavirus in Western Europe [36]. As an integrated analysis tool of spillover surveillance programs, Microseek has proved to be effective in detecting many novel or distant viruses from wildlife and arthropods, among which a novel polycipivirus from *Pteropus lylei* bats [37], a novel reovirus in Laotian batflies [38], a novel picornavirus-like virus with an unusual genome organization in frugivorous bats in Cambodia [39] and, remarkably, bat coronaviruses closely related to SARS-CoV-2 and infectious for human cells in Laos [40]. Microseek was used to describe the diversity of viruses present in ticks as exemplified by the exploration of the Chuviridae, Phenuiviridae and Flaviviridae families identified from Caribbean *Amblyomma* and *Rhipicephalus* ticks [41], and was useful at increasing knowledge on the host range, genetic diversity, and geographical distribution of Jingmenviruses [42]. Microseek was also used to detect viruses with potential relevance for public health found in ticks from Eastern Romania [43], in particular in the Danube Delta [30], and to track silent spillovers before emergence at the tick/human interface in Thailand [44]. As of July 2022, and over the past year, RVDB-prot, the core database of Microseek, was downloaded more than 10,400 times, showing the interest of the metagenomics research community in having a comprehensive and curated viral protein database.

A smart feature of Microseek is its dynamic HTML interface allowing easy data mining by a biologist. The interplay between a graphical interface and a sortable data table provides both a synthesis view and a direct access to the sequences and their associated GenBank and PubMed entries. The standalone web-page format facilitates sharing of results. This interface thus favors viral discovery and collaborative activities. Nevertheless, a simplified graphical interface or a simplified B1 database retaining only viruses of known medical interest could be proposed to medical virology laboratories for diagnostic purposes.

Microseek was developed in the context of an institutional cluster providing abundant resources and was therefore optimized for accuracy. In order to make it compatible with more modest computational facilities, including desktop computers, efforts could be made on reducing computation times for some steps. For instance, B3 is the most CPU consuming step and since it aims at discarding non-coding sequences, it could be replaced by a less sensitive algorithm. Diamond, used in Microseek and integrated as a scalability upgrade in CZ ID pipeline Version 7.0, has shown its huge gain in performance on benchmark studies, but other similar blast-like algorithms were also proposed such as MMseqs2 [45] and claim the same level of precision. In addition, some scripts could be parallelized to take advantage of a DataBase Management System (DBMS) faster than SQLite (on which rely some databases for annotation), such as PostgreSQL. We plan to offer such upgrades in the future to facilitate the broad adoption of Microseek by the community.

## 5. Conclusions

Microseek is part of the metagenomic community’s effort to develop accurate and easy-to-use tools for viral diagnostic and virus discovery. Its protein-based approach showed performance comparable to that of the state-of-the-art and cloud-based CZ ID software for the detection of known viruses. Microseek also facilitates the detection of distant pseudo-viral sequences in matrices with a high non-human content, such as plasma, by limiting the reporting of non-relevant hits. Microseek has demonstrated its value for medical diagnosis and is at the heart of research works on viral emergence. The software is supported through regular updates of its container and of the RVDB-prot database.

## Figures and Tables

**Table 1:** Detection of known viruses. The table represents the detection of spike vs. detected sequences of the six known viruses at dilution 1, dilution 10 and dilution 100 in the Tissue and Plasma experiments. sp.: species.

			TISSUES						PLASMA					
			*Spike*		*Detection*				*Spike*		*Detection*			
			Number of sequences	Horizontal coverage (%)	Horizontal coverage (%)		Taxonomic precision (family, genus, sp)		Number of sequences	Horizontal coverage	Horizontal coverage (%)		Taxonomic precision (family, genus, sp)	
**Color code used in Figure 2**					Microseek	CZ ID	Microseek	CZ ID			Microseek	CZ ID	Microseek	CZ ID
			**[dilution 1]**											
	ssDNA	Parvovirus B19	158	99.8	92.7	99.0	sp.	sp.	533	99.8	90.8	99.0	sp.	sp.
	dsDNA	Epstein–Barr virus	500	7.5	5.7	6.5	sp.	sp.	3650	96.3	79.7	51.0	sp.	sp.
	ssRNA+	Coxsackievirus B6	171	94.8	93.8	94.5	sp.	sp.	667	99.6	99.3	96.4	sp.	sp.
	ssRNA−	Respiratory syncytial virus	107	62.8	59.5	57.4	sp.	sp.	67	51.1	49.1	18.1	sp.	sp.
	dsRNA	Reovirus 1 (10 segments)	1404	98.3	97.7	98.3	sp.	sp.	3786	97.9	97.4	92.7	sp.	sp.
	Retrovirus	HIV-1	160	59.0	50.8	55.8	sp.	sp.	556	99.7	55.3	93.0	sp.	sp.
			**[dilution 10]**											
	ssDNA	Parvovirus B19	16	35.1	30.4	29.7	sp.	sp.	53	78.7	69.5	34.4	sp.	sp.
	dsDNA	Epstein–Barr virus	49	2.0	1.7	1.9	sp.	sp.	365	27.1	19.3	7.8	sp.	sp.
	ssRNA+	Coxsackievirus B6	18	30.7	27.1	27.0	sp.	sp.	67	78.5	72.1	30.3	sp.	sp.
	ssRNA−	Respiratory syncytial virus	11	10.4	9.4	10.4	sp.	sp.	7	6.9	6.9	1.0	sp.	sp.
	dsRNA	Reovirus 1 (10 segments)	138	56.9	56.5	55.5	sp.	sp.	379	75.9	75.2	38.7	sp.	sp.
	Retrovirus	HIV-1	16	22.5	16.4	19.2	sp.	sp.	56	63.5	25.9	27.4	sp.	sp.
			**[dilution 100]**											
	ssDNA	Parvovirus B19	3	7.3	2.7	7.3	genus	sp.	5	13.4	10.7	0.0	sp.	n.a.
	dsDNA	Epstein–Barr virus	5	0.3	0.3	0.3	sp.	sp.	36	3.1	2.5	0.8	sp.	sp.
	ssRNA+	Coxsackievirus B6	3	5.6	2.0	5.6	sp.	sp.	7	14.2	14.2	2.0	sp.	sp.
	ssRNA−	Respiratory syncytial virus	3	3.0	3.0	3.0	sp.	sp.	1	1.0	1.0	1.0	sp.	sp.
	dsRNA	Reovirus 1 (10 segments)	30	16.1	16.1	15.8	sp.	sp.	37	20.4	20.4	6.4	sp.	sp.
	Retrovirus	HIV-1	2	3.3	1.6	3.3	sp.	sp.	6	9.8	1.6	3.3	genus	sp.

**Table 2:** Detection of Neopneumoviruses. The table represents the detection of spike vs. detected sequences of the three Neopneumoviruses at dilution 1 and dilution 10 in the Tissue and Plasma experiments. n.a.: not applicable.

		TISSUES						PLASMA					
		*Spike*		*Detection*				*Spike*		*Detection*			
		Number of sequences	Horizontal coverage (%)	Horizontal coverage (%)		Taxonomic precision (family, genus, sp)		Number of sequences	Horizontal coverage	Horizontal coverage (%)		Taxonomic precision (family, genus, sp)	
**Color code used in Figure 4**				Microseek	CZ ID	Microseek	CZ ID			Microseek	CZ ID	Microseek	CZ ID
		**[dilution 1]**											
	Neopneumovirus-1	107	81.6	73.3	71.5	**family** *(Orthopneumovirus: Human, Bovine, Murine, + Ovine respiratory syncytial virus + Unassigned Pneumoviridae)*	**family** *(Orthopneumovirus: Human, Murine, Bovine + Non-genus-specific reads in the family Pneumoviridae)*	67	52.2	47.7	19.4	**family** *(Orthopneumovirus: Human, Bovine, Swine, Murine + Unassigned Pneumoviridae)*	**genus** *(Orthopneumovirus: Human, Murine, Bovine)*
	Neopneumovirus-2	107	70.5	51.6	56.6	**family** *(Orthopneumovirus: Human, Bovine, Murine, + Unassigned Pneumoviridae)*	**family** *(Orthopneumovirus: Human, Murine, Bovine + Non-genus-specific reads in the family Pneumoviridae)*	67	56.1	38.1	18.4	**family** *(Orthopneumovirus: Human, Bovine, Swine, Murine + Ovine Respiratory Syncytial Virus + Unassigned Pneumoviridae)*	**genus** *(Orthopneumovirus: Human, Murine, Bovine)*
	Neopneumovirus-3	107	70.1	20.6	34.0	**genus** *(Orthopneumovirus: Human, Bovine)*	**family** *(Orthopneumovirus: Human, Murine, Bovine + Non-genus-specific reads in the family Pneumoviridae)*	67	52.5	14.3	11.8	**species** *(Human respiratory syncytial virus B, Human orthopneumovirus)*	**genus** *(Orthopneumovirus: Human, Bovine)*
		**[dilution 10]**											
	Neopneumovirus-1	11	12.1	9.7	10.9	**family** *(Orthopneumovirus: Human, Bovine + Unassigned Pneumoviridae)*	**family** *(Orthopneumovirus: Human, Bovine + Non-genus-specific reads in the family Pneumoviridae)*	7	8.4	7.2	1.2	**genus** *(Orthopneumovirus: Human, Bovine, Swine, Unassigned)*	**species** *(Human orthopneumovirus)*
	Neopneumovirus-2	11	12.9	4.8	10.5	**genus** *(Orthopneumovirus: Human, Bovine)*	**family** *(Orthopneumovirus: Human, Bovine + Non-genus-specific reads in the family Pneumoviridae)*	7	8.4	3.6	3.6	**species** *(Human orthopneumovirus)*	**species** *(Human orthopneumovirus)*
	Neopneumovirus-3	11	12.1	0.0	3.6	n.a.	**family** *(Orthopneumovirus: Human, Bovine + Non-genus-specific reads in the family Pneumoviridae)*	7	7.6	2.7	1.2	**genus** *(Orthopneumovirus: Human, Bovine)*	**family** *(Non-genus-specific reads in the family Pneumoviridae)*

## Data Availability

The data presented in this study are available online at Zenodo (https://doi.org/10.5281/zenodo.6937000, accessed on 28 July 2022; Creative Commons Attribution 4.0 International License).

## Supplementary Materials

The following supporting information can be downloaded at: https://www.mdpi.com/article/10.3390/v14091990/s1, Figure S1: Mapping of spiked and detected sequences. Table S1: Rules followed for the generation of spiked datasets; Table S2: Id matrices between Neopneumoviruses-1 -2 -3 and HRSV-A; Table S3: Datasets analyzed in the absence of additional in-silico spike; Table S4: Residual hits following the analysis of the Plasma datasets spiked with known viral sequences; Table S5: Residual hits following the analysis of the Plasma datasets spiked with Neopneumoviruses sequences; Table S6: HMM results; Table S7: Results of classifiers based on kmer composition (Kraken2 and Centrifuge) or on predefined markers (Metaphlan 2).

## References

[1] Filkins L.M., Bryson A.L., Miller S.A., Mitchell S.L. (2020). Navigating Clinical Utilization of Direct-from-Specimen Metagenomic Pathogen Detection: Clinical Applications, Limitations, and Testing Recommendations. Clin. Chem..

[2] Center for Devices and Radiological Health Considerations for Design, Development, and Analytical Validation of Next Generation Sequencing (NGS)-Based In Vitro Diagnostics (IVDs) Intended to Aid in the Diagnosis of Suspected Germline Diseases. https://www.fda.gov/regulatory-information/search-fda-guidance-documents/considerations-design-development-and-analytical-validation-next-generation-sequencing-ngs-based.

[3] López-Labrador F.X., Brown J.R., Fischer N., Harvala H., Van Boheemen S., Cinek O., Sayiner A., Madsen T.V., Auvinen E., Kufner V. (2021). Recommendations for the Introduction of Metagenomic High-Throughput Sequencing in Clinical Virology, Part I: Wet Lab Procedure. J. Clin. Virol..

[4] De Vries J.J.C., Brown J.R., Couto N., Beer M., Le Mercier P., Sidorov I., Papa A., Fischer N., Oude Munnink B.B., Rodriquez C. (2021). Recommendations for the Introduction of Metagenomic Next-Generation Sequencing in Clinical Virology, Part II: Bioinformatic Analysis and Reporting. J. Clin. Virol..

[5] Khan A.S., Blümel J., Deforce D., Gruber M.F., Jungbäck C., Knezevic I., Mallet L., Mackay D., Matthijnssens J., O’Leary M. (2020). Report of the Second International Conference on next Generation Sequencing for Adventitious Virus Detection in Biologics for Humans and Animals. Biologicals.

[6] Ng S.H., Braxton C., Eloit M., Feng S.F., Fragnoud R., Mallet L., Mee E.T., Sathiamoorthy S., Vandeputte O., Khan A.S. (2018). Current Perspectives on High-Throughput Sequencing (HTS) for Adventitious Virus Detection: Upstream Sample Processing and Library Preparation. Viruses.

[7] Lambert C., Braxton C., Charlebois R.L., Deyati A., Duncan P., La Neve F., Malicki H.D., Ribrioux S., Rozelle D.K., Michaels B. (2018). Considerations for Optimization of High-Throughput Sequencing Bioinformatics Pipelines for Virus Detection. Viruses.

[8] Naccache S.N., Federman S., Veeraraghavan N., Zaharia M., Lee D., Samayoa E., Bouquet J., Greninger A.L., Luk K.-C., Enge B. (2014). A Cloud-Compatible Bioinformatics Pipeline for Ultrarapid Pathogen Identification from next-Generation Sequencing of Clinical Samples. Genome Res..

[9] Kostic A.D., Ojesina A.I., Pedamallu C.S., Jung J., Verhaak R.G.W., Getz G., Meyerson M. (2011). PathSeq: Software to Identify or Discover Microbes by Deep Sequencing of Human Tissue. Nat. Biotechnol..

[10] Walker M.A., Pedamallu C.S., Ojesina A.I., Bullman S., Sharpe T., Whelan C.W., Meyerson M. (2018). GATK PathSeq: A Customizable Computational Tool for the Discovery and Identification of Microbial Sequences in Libraries from Eukaryotic Hosts. Bioinformatics.

[11] Flygare S., Simmon K., Miller C., Qiao Y., Kennedy B., Di Sera T., Graf E.H., Tardif K.D., Kapusta A., Rynearson S. (2016). Taxonomer: An Interactive Metagenomics Analysis Portal for Universal Pathogen Detection and Host MRNA Expression Profiling. Genome Biol..

[12] Kalantar K.L., Carvalho T., de Bourcy C.F.A., Dimitrov B., Dingle G., Egger R., Han J., Holmes O.B., Juan Y.-F., King R. (2020). IDseq-An Open Source Cloud-Based Pipeline and Analysis Service for Metagenomic Pathogen Detection and Monitoring. Gigascience.

[13] Goodacre N., Aljanahi A., Nandakumar S., Mikailov M., Khan A.S. (2018). A Reference Viral Database (RVDB) To Enhance Bioinformatics Analysis of High-Throughput Sequencing for Novel Virus Detection. mSphere.

[14] Bigot T., Temmam S., Pérot P., Eloit M. (2019). RVDB-Prot, a Reference Viral Protein Database and Its HMM Profiles. F1000Res.

[15] Criscuolo A., Brisse S. (2013). AlienTrimmer: A Tool to Quickly and Accurately Trim off Multiple Short Contaminant Sequences from High-Throughput Sequencing Reads. Genomics.

[16] BBMap. https://sourceforge.net/projects/bbmap/.

[17] Crusoe M.R., Alameldin H.F., Awad S., Boucher E., Caldwell A., Cartwright R., Charbonneau A., Constantinides B., Edvenson G., Fay S. (2015). The Khmer Software Package: Enabling Efficient Nucleotide Sequence Analysis. F1000Res.

[18] Li D., Liu C.-M., Luo R., Sadakane K., Lam T.-W. (2015). MEGAHIT: An Ultra-Fast Single-Node Solution for Large and Complex Metagenomics Assembly via Succinct de Bruijn Graph. Bioinformatics.

[19] Langmead B., Salzberg S.L. (2012). Fast Gapped-Read Alignment with Bowtie 2. Nat. Methods.

[20] TranslateReads.Py. /articles/code/translateReads_py/7588592/1.

[21] Buchfink B., Xie C., Huson D.H. (2015). Fast and Sensitive Protein Alignment Using DIAMOND. Nat. Methods.

[22] Meyers G., Rümenapf T., Tautz N., Dubovi E.J., Thiel H.-J., Liess B., Moennig V., Pohlenz J., Trautwein G. (1991). Insertion of Cellular Sequences in the Genome of Bovine Viral Diarrhea Virus. Ruminant Pestivirus Infections.

[23] Ondov B.D., Bergman N.H., Phillippy A.M. (2011). Interactive Metagenomic Visualization in a Web Browser. BMC Bioinform..

[24] Asplund M., Kjartansdóttir K.R., Mollerup S., Vinner L., Fridholm H., Herrera J.a.R., Friis-Nielsen J., Hansen T.A., Jensen R.H., Nielsen I.B. (2019). Contaminating Viral Sequences in High-Throughput Sequencing Viromics: A Linkage Study of 700 Sequencing Libraries. Clin. Microbiol. Infect..

[25] Zong C., Lu S., Chapman A.R., Xie X.S. (2012). Genome-Wide Detection of Single-Nucleotide and Copy-Number Variations of a Single Human Cell. Science.

[26] Huang W., Li L., Myers J.R., Marth G.T. (2012). ART: A next-Generation Sequencing Read Simulator. Bioinformatics.

[27] Pérot P., Bielle F., Bigot T., Foulongne V., Bolloré K., Chrétien D., Gil P., Gutiérrez S., L’Ambert G., Mokhtari K. (2020). Identification of Umbre Orthobunyavirus as a Novel Zoonotic Virus Responsible for Lethal Encephalitis in 2 French Patients with Hypogammaglobulinemia. Clin. Infect. Dis..

[28] Regnault B., Bigot T., Ma L., Pérot P., Temmam S., Eloit M. (2021). Deep Impact of Random Amplification and Library Construction Methods on Viral Metagenomics Results. Viruses.

[29] Köster J., Rahmann S. (2012). Snakemake—A Scalable Bioinformatics Workflow Engine. Bioinformatics.

[30] Bratuleanu B.E., Temmam S., Munier S., Chrétien D., Bigot T., van der Werf S., Savuta G., Eloit M. (2022). Detection of Phenuiviridae, Chuviridae Members, and a Novel Quaranjavirus in Hard Ticks From Danube Delta. Front. Vet. Sci..

[31] Zhang Y.-Z., Chen Y.-M., Wang W., Qin X.-C., Holmes E.C. (2019). Expanding the RNA Virosphere by Unbiased Metagenomics. Annu. Rev. Virol..

[32] Wood D.E., Lu J., Langmead B. (2019). Improved Metagenomic Analysis with Kraken 2. Genome Biol..

[33] Kim D., Song L., Breitwieser F.P., Salzberg S.L. (2016). Centrifuge: Rapid and Sensitive Classification of Metagenomic Sequences. Genome Res..

[34] Truong D.T., Franzosa E.A., Tickle T.L., Scholz M., Weingart G., Pasolli E., Tett A., Huttenhower C., Segata N. (2015). MetaPhlAn2 for Enhanced Metagenomic Taxonomic Profiling. Nat. Methods.

[35] Capra J.A., Singh M. (2007). Predicting Functionally Important Residues from Sequence Conservation. Bioinformatics.

[36] Regnault B., Evrard B., Plu I., Dacheux L., Troadec E., Cozette P., Chrétien D., Duchesne M., Jean-Michel V., Jamet A. (2021). First Case of Lethal Encephalitis in Western Europe Due to European Bat Lyssavirus Type 1. Clin. Infect. Dis..

[37] Temmam S., Hul V., Bigot T., Hoem T., Gorman C., Duong V., Dussart P., Cappelle J., Eloit M. (2019). A Novel Polycipiviridae Virus Identified in Pteropus Lylei Stools. Microbiol. Resour. Announc..

[38] Temmam S., Vongphayloth K., Hertz J.C., Sutherland I., Douangboubpha B., Grandadam M., Bigot T., Brey P.T., Eloit M. (2019). Six Nearly Complete Genome Segments of a Novel Reovirus Identified in Laotian Batflies. Microbiol. Resour. Announc..

[39] Temmam S., Hul V., Bigot T., Bessaud M., Chrétien D., Hoem T., Gorman C., Duong V., Dussart P., Cappelle J. (2020). Whole Genome Sequencing and Phylogenetic Characterization of a Novel Bat-Associated Picornavirus-like Virus with an Unusual Genome Organization. Infect. Genet. Evol..

[40] Temmam S., Vongphayloth K., Baquero E., Munier S., Bonomi M., Regnault B., Douangboubpha B., Karami Y., Chrétien D., Sanamxay D. (2022). Bat Coronaviruses Related to SARS-CoV-2 and Infectious for Human Cells. Nature.

[41] Gondard M., Temmam S., Devillers E., Pinarello V., Bigot T., Chrétien D., Aprelon R., Vayssier-Taussat M., Albina E., Eloit M. (2020). RNA Viruses of Amblyomma Variegatum and Rhipicephalus Microplus and Cattle Susceptibility in the French Antilles. Viruses.

[42] Temmam S., Bigot T., Chrétien D., Gondard M., Pérot P., Pommelet V., Dufour E., Petres S., Devillers E., Hoem T. (2019). Insights into the Host Range, Genetic Diversity, and Geographical Distribution of Jingmenviruses. mSphere.

[43] Bratuleanu B.E., Temmam S., Chrétien D., Regnault B., Pérot P., Bouchier C., Bigot T., Savuța G., Eloit M. (2021). The Virome of Rhipicephalus, Dermacentor and Haemaphysalis Ticks from Eastern Romania Includes Novel Viruses with Potential Relevance for Public Health. Transbound Emerg. Dis..

[44] Temmam S., Chrétien D., Bigot T., Dufour E., Petres S., Desquesnes M., Devillers E., Dumarest M., Yousfi L., Jittapalapong S. (2019). Monitoring Silent Spillovers Before Emergence: A Pilot Study at the Tick/Human Interface in Thailand. Front. Microbiol..

[45] Steinegger M., Söding J. (2017). MMseqs2 Enables Sensitive Protein Sequence Searching for the Analysis of Massive Data Sets. Nat. Biotechnol..

