# Supplementary material for: Microseek: A Protein-Based Metagenomic Pipeline for Virus Diagnostic and Discovery
Source: Viruses. 2022 Sep 8;14(9):1990. doi: 10.3390/v14091990 (PMC9500916; doi:10.3390/v14091990)
Supplement: Supplementary file 1 [file viruses-14-01990-s001.zip › FigureS1.pptx]

## Slide 1
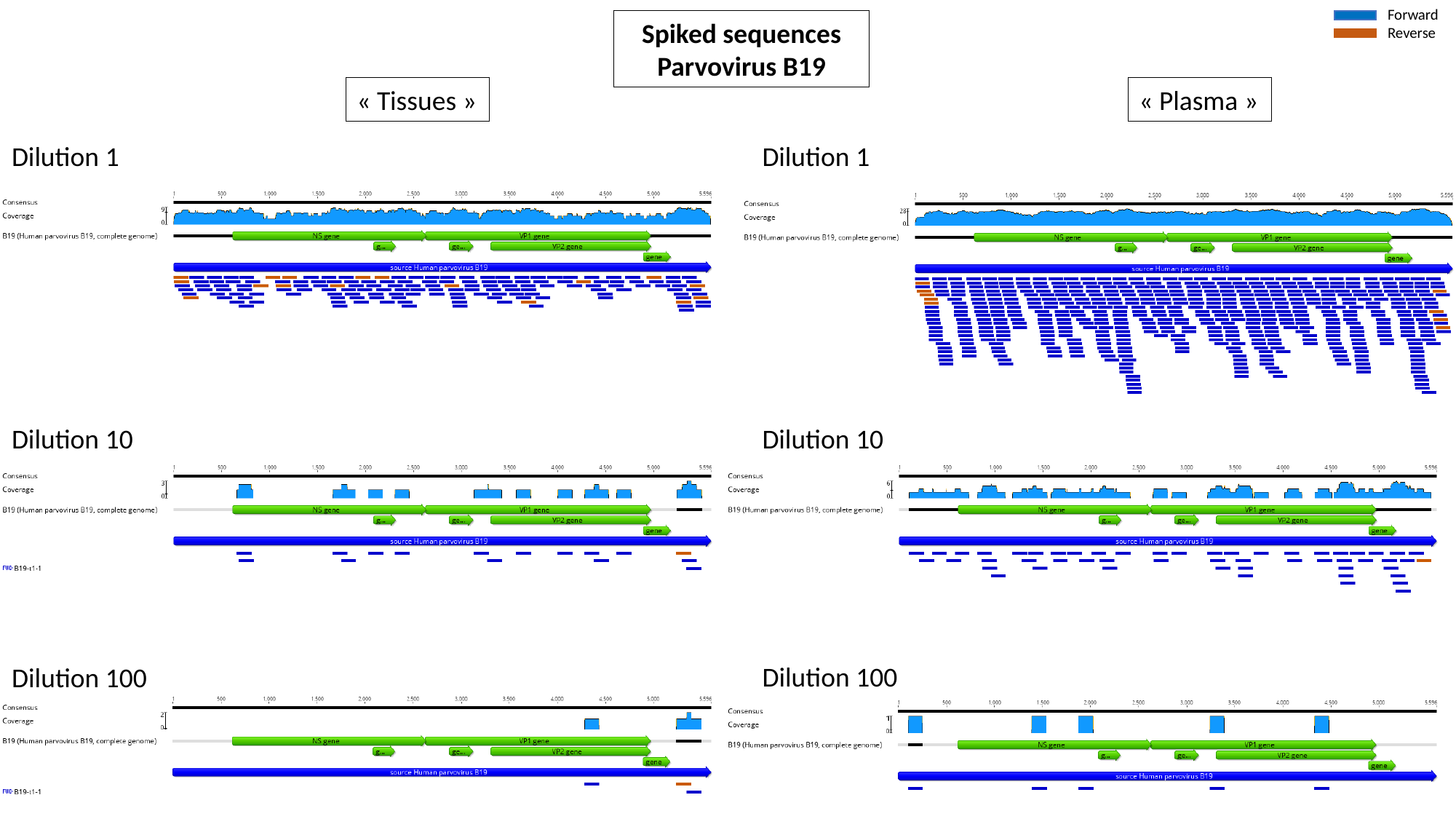

Forward
Reverse
Spiked sequences
Parvovirus B19
« Tissues »
« Plasma »
Dilution 1
Dilution 1
Dilution 10
Dilution 10
Dilution 100
Dilution 100

## Slide 2
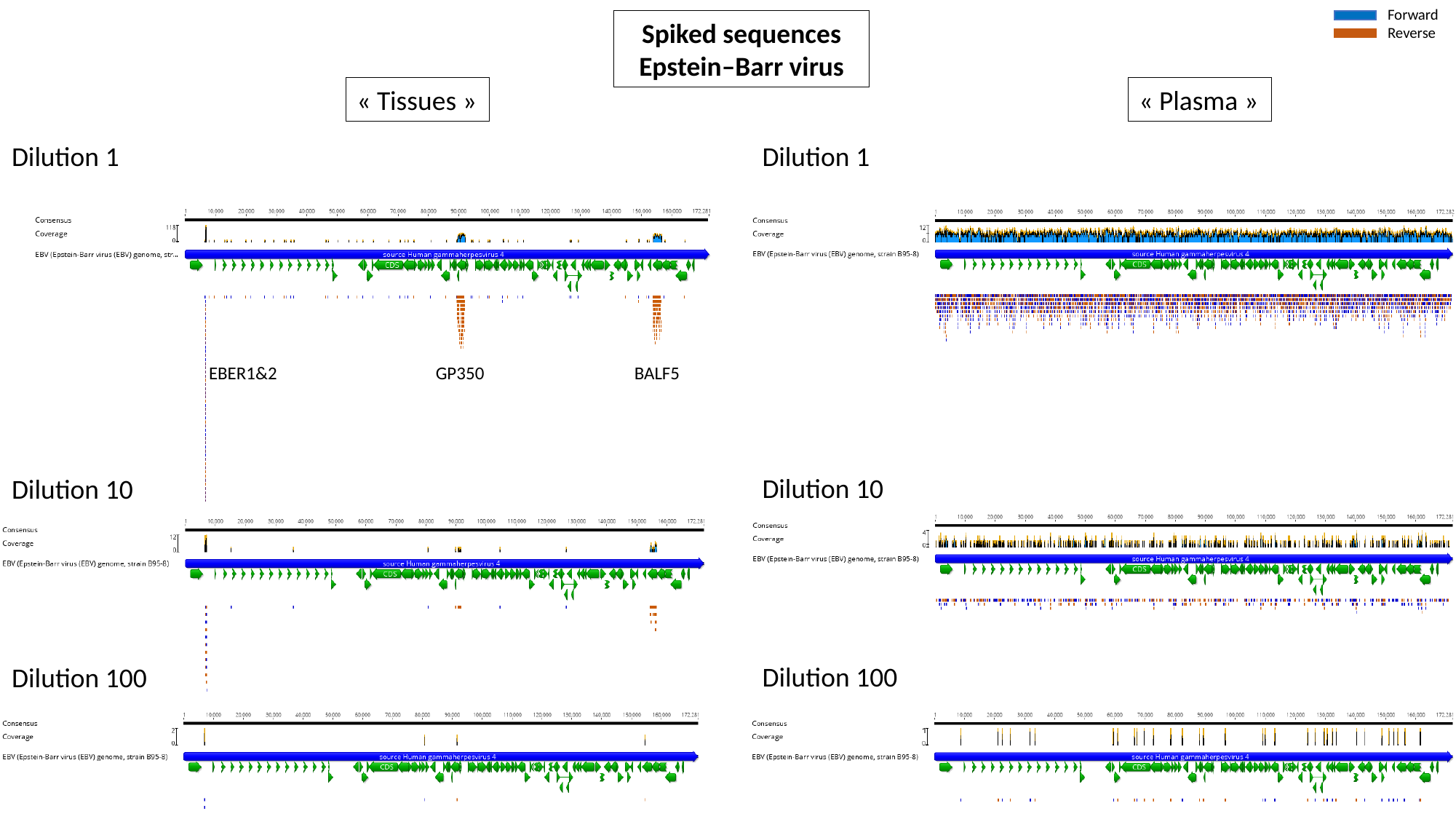

Forward
Reverse
Spiked sequences
Epstein–Barr virus
« Tissues »
« Plasma »
Dilution 1
Dilution 1
EBER1&2
GP350
BALF5
Dilution 10
Dilution 10
Dilution 100
Dilution 100

## Slide 3
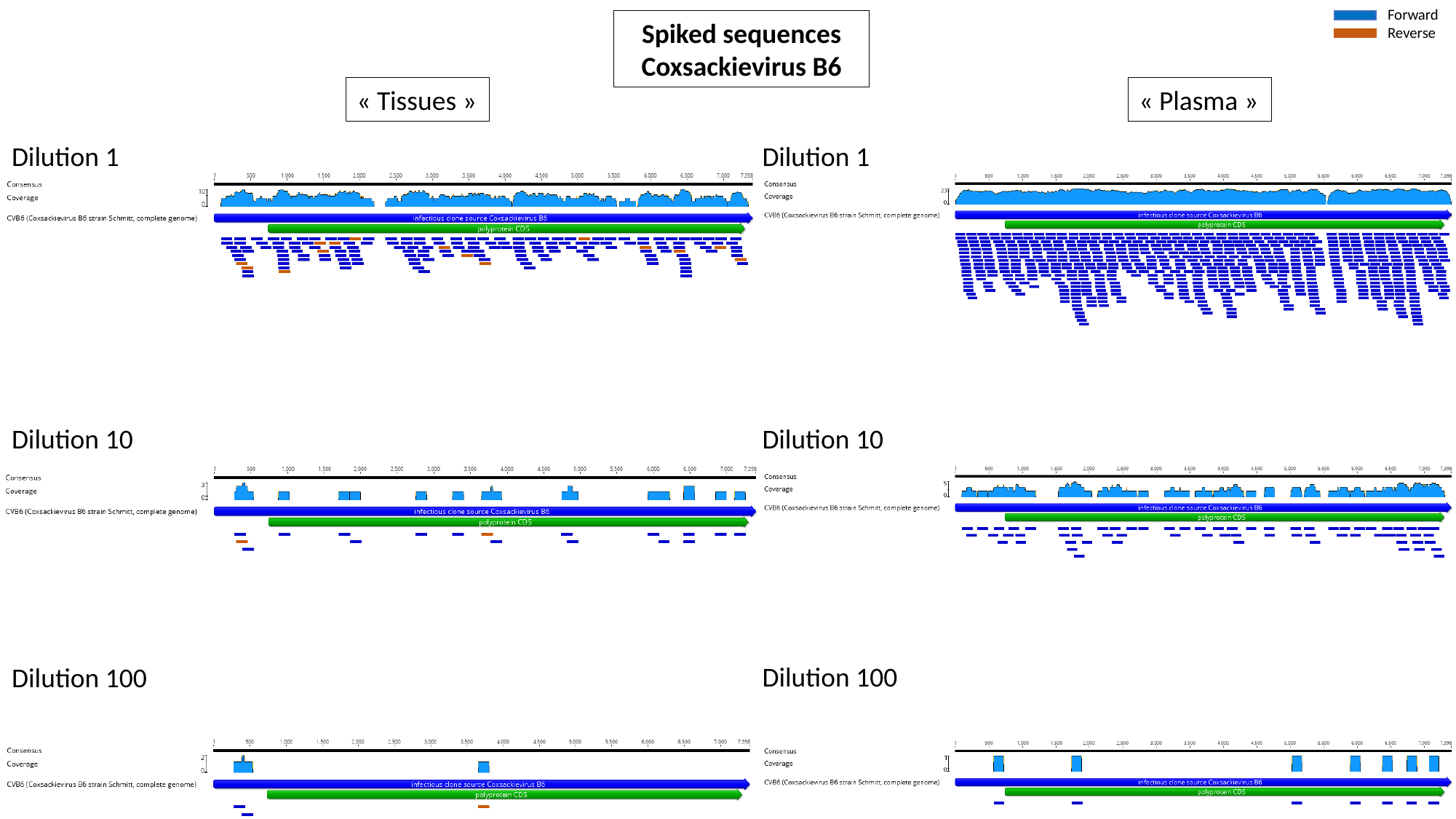

Forward
Reverse
Spiked sequences
Coxsackievirus B6
« Tissues »
« Plasma »
Dilution 1
Dilution 1
Dilution 10
Dilution 10
Dilution 100
Dilution 100

## Slide 4
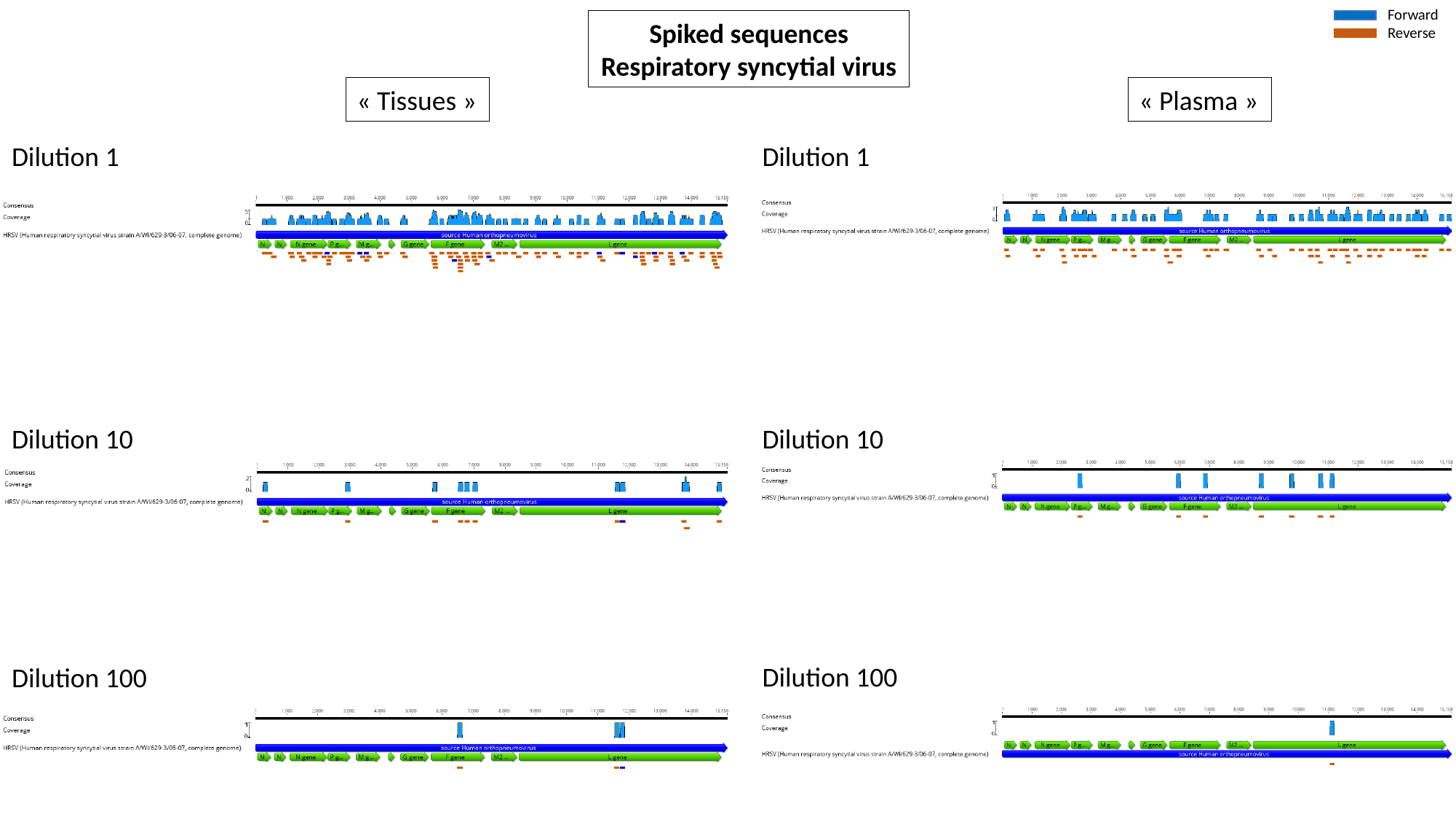

Forward
Reverse
Spiked sequences
Respiratory syncytial virus
« Tissues »
« Plasma »
Dilution 1
Dilution 1
Dilution 10
Dilution 10
Dilution 100
Dilution 100

## Slide 5
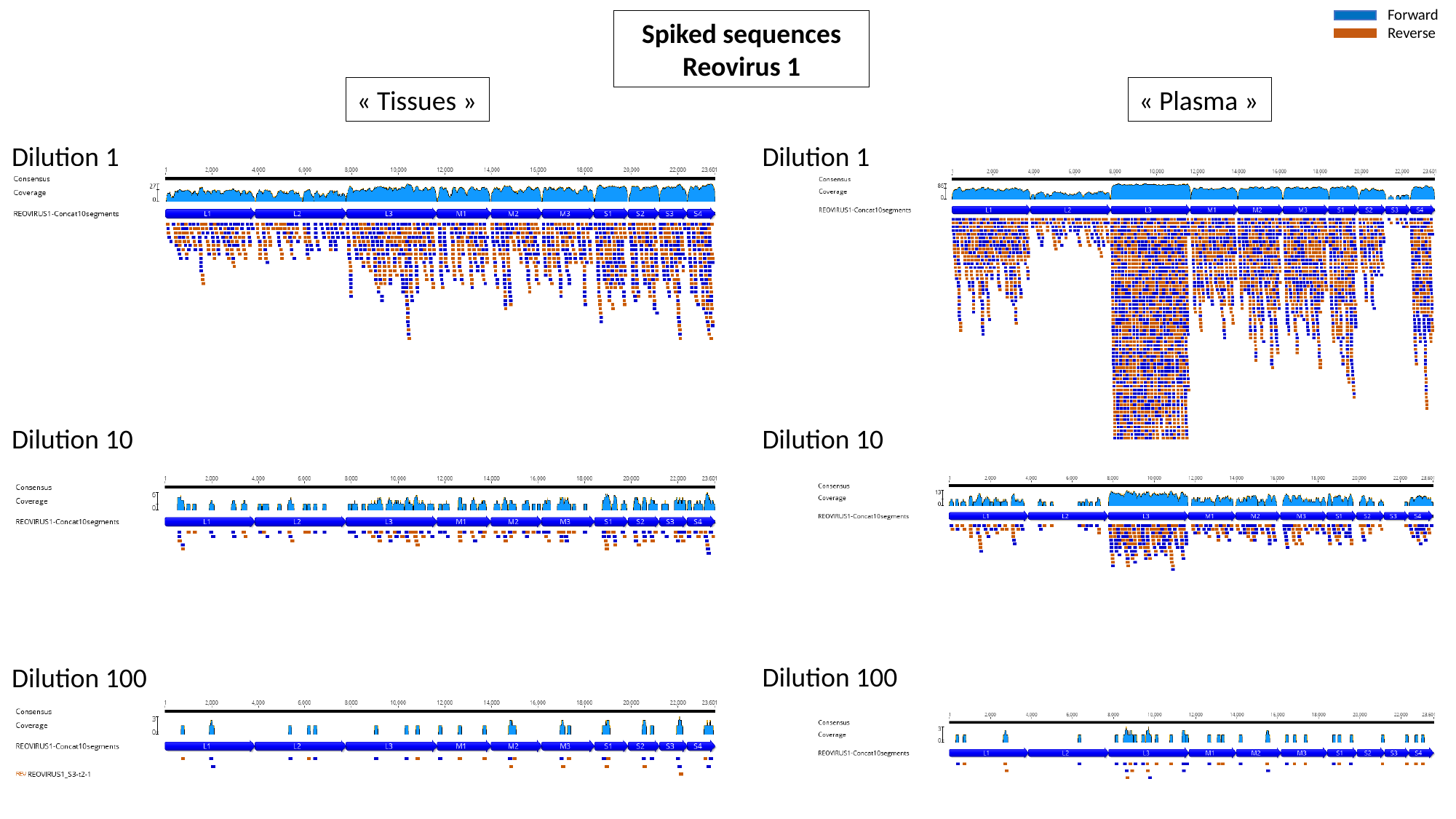

Forward
Reverse
Spiked sequences
Reovirus 1
« Tissues »
« Plasma »
Dilution 1
Dilution 1
Dilution 10
Dilution 10
Dilution 100
Dilution 100

## Slide 6
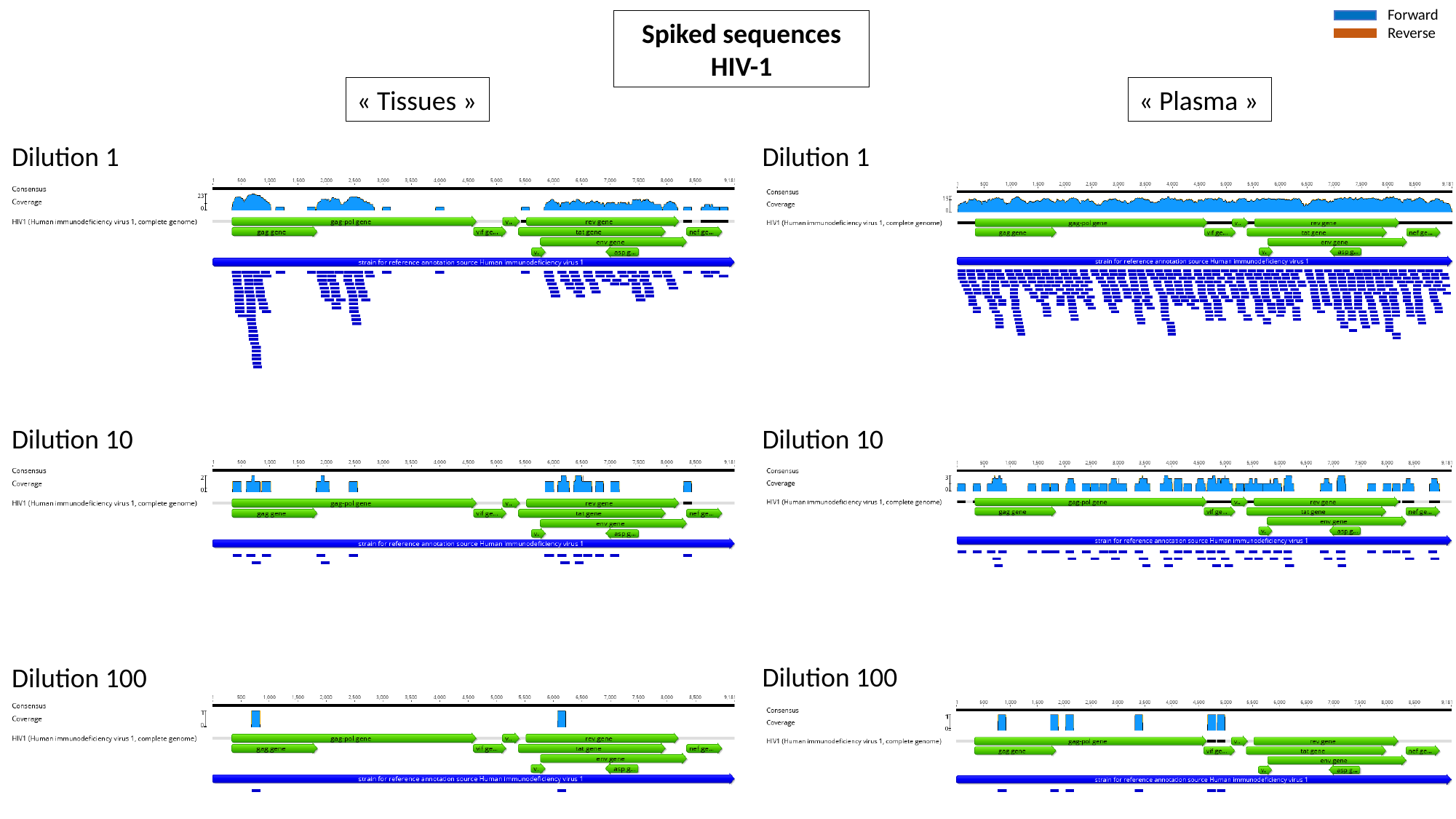

Forward
Reverse
Spiked sequences
HIV-1
« Tissues »
« Plasma »
Dilution 1
Dilution 1
Dilution 10
Dilution 10
Dilution 100
Dilution 100

## Slide 7
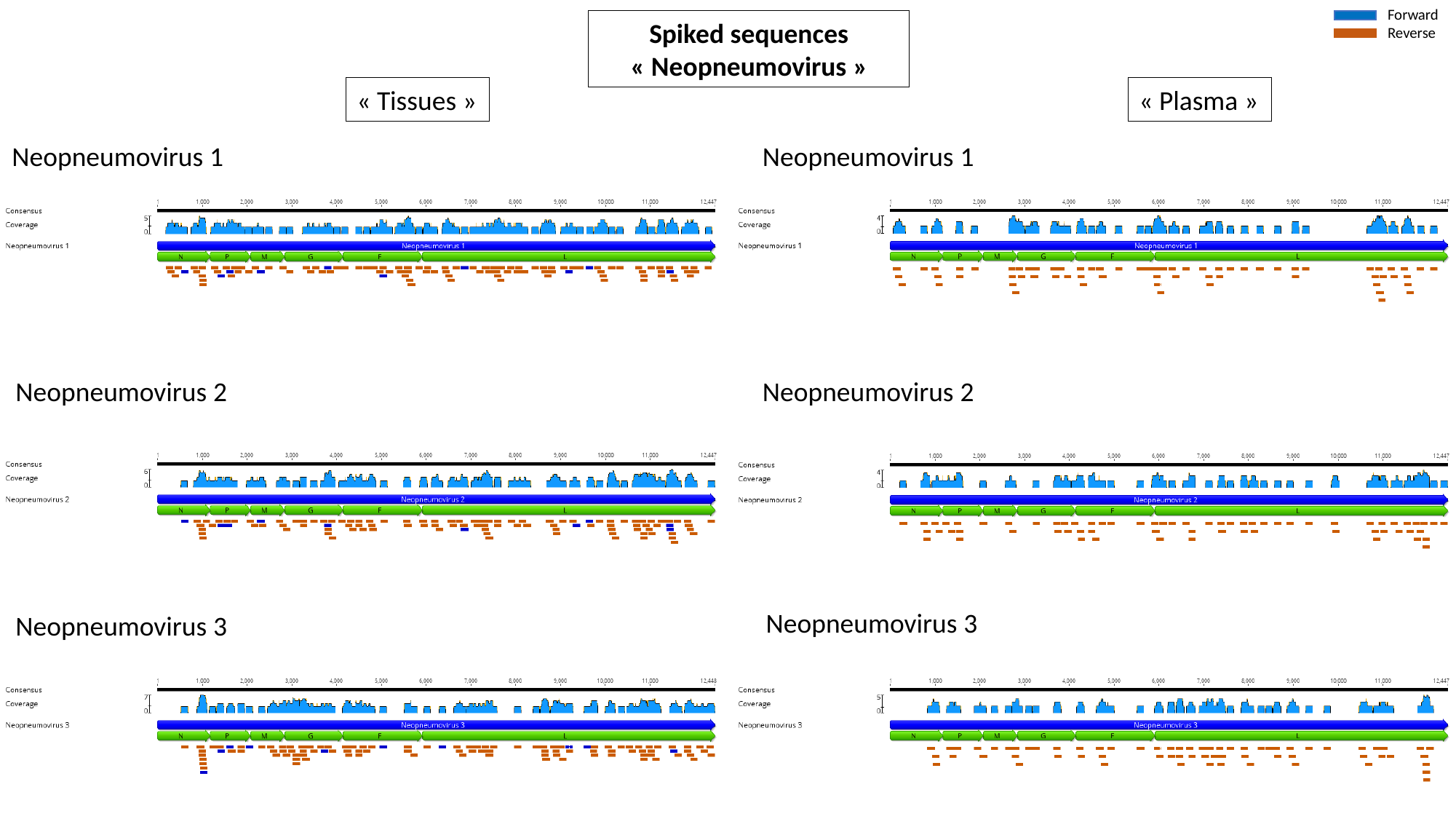

Forward
Reverse
Spiked sequences
« Neopneumovirus »
« Tissues »
« Plasma »
Neopneumovirus 1
Neopneumovirus 1
Neopneumovirus 2
Neopneumovirus 2
Neopneumovirus 3
Neopneumovirus 3

## Slide 8
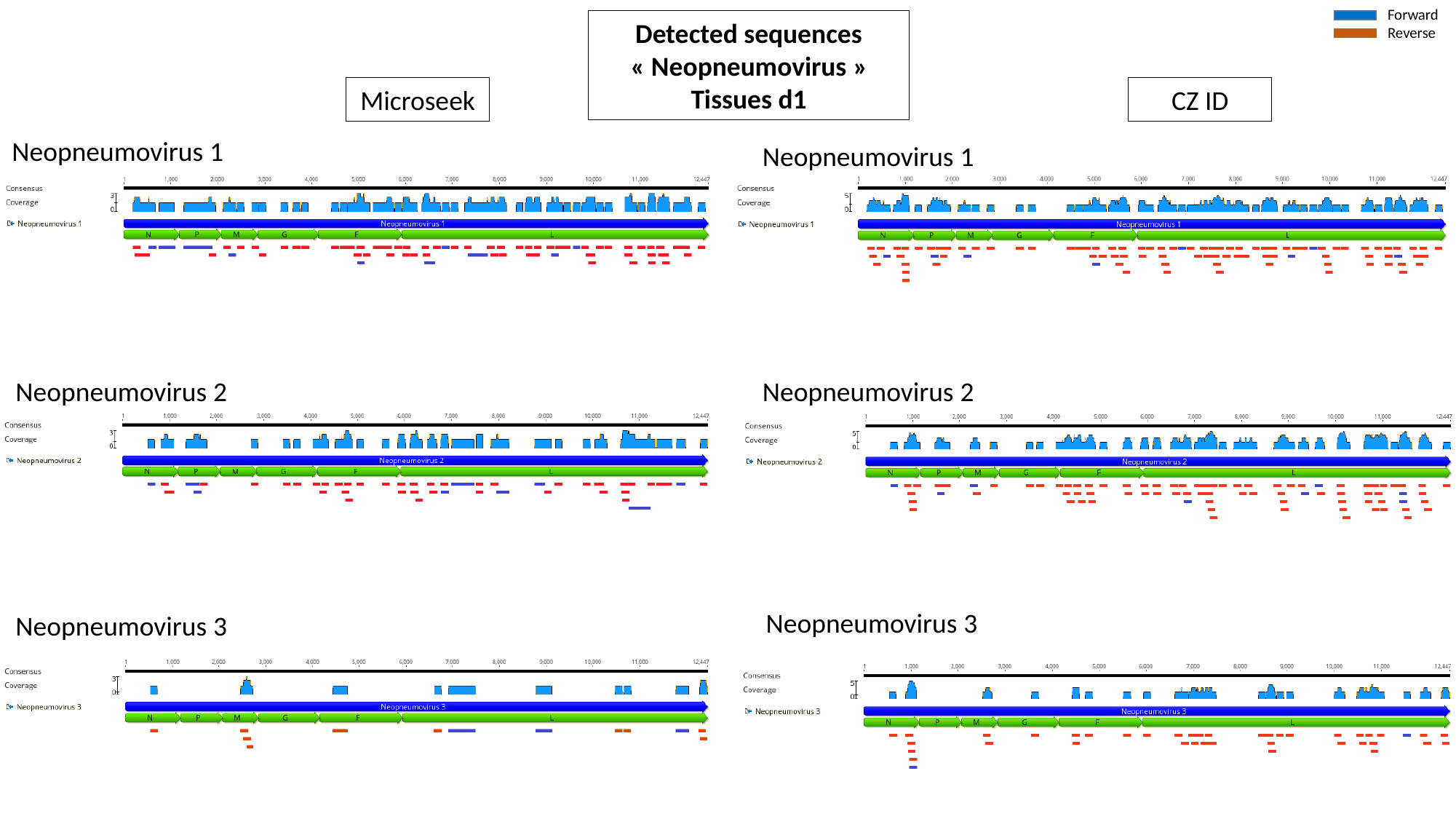

Forward
Reverse
Detected sequences
« Neopneumovirus »
Tissues d1
Microseek
CZ ID
Neopneumovirus 1
Neopneumovirus 1
Neopneumovirus 2
Neopneumovirus 2
Neopneumovirus 3
Neopneumovirus 3

## Slide 9
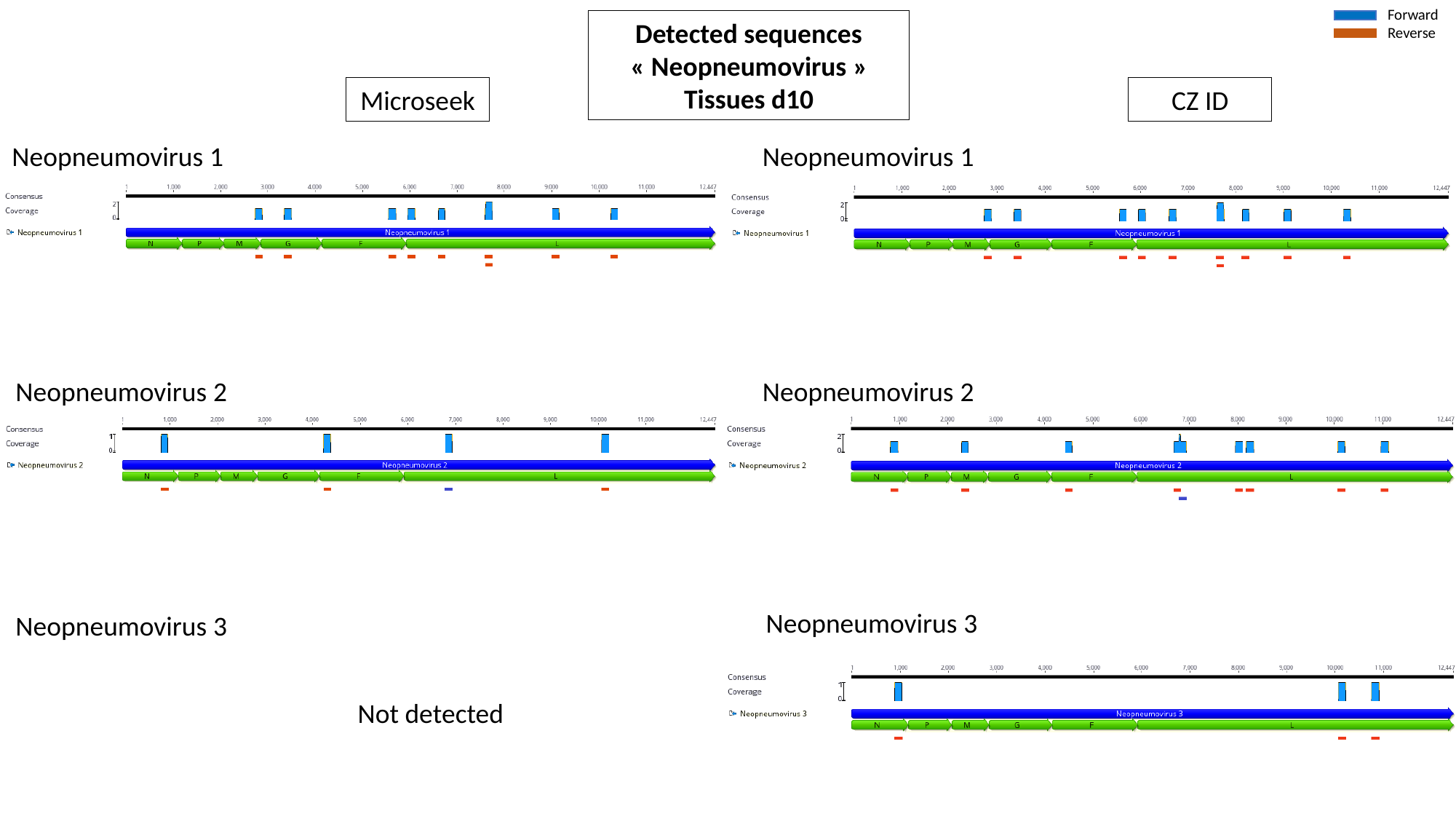

Forward
Reverse
Detected sequences
« Neopneumovirus »
Tissues d10
Microseek
CZ ID
Neopneumovirus 1
Neopneumovirus 1
Neopneumovirus 2
Neopneumovirus 2
Neopneumovirus 3
Neopneumovirus 3
Not detected

## Slide 10
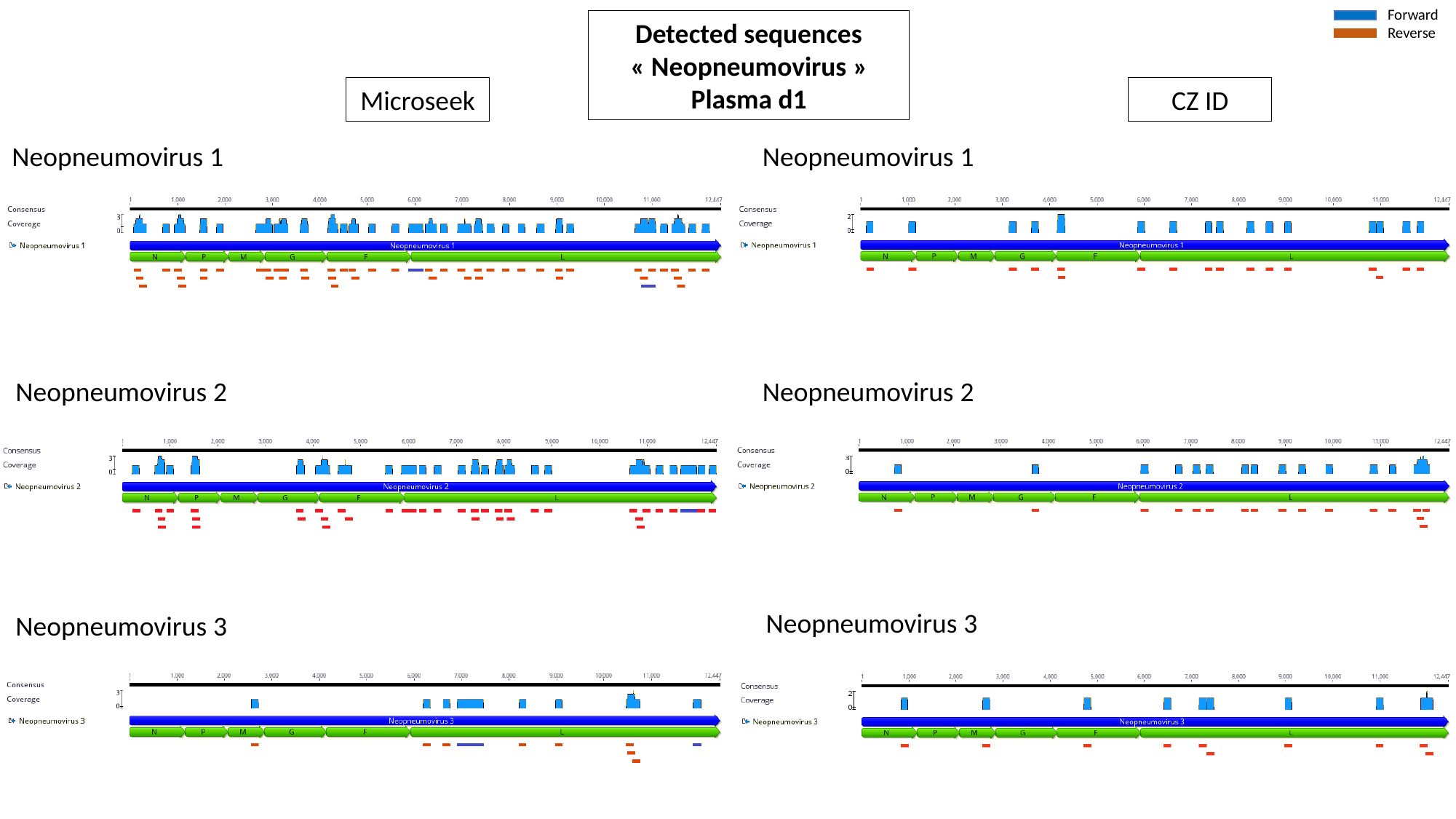

Forward
Reverse
Detected sequences
« Neopneumovirus »
Plasma d1
Microseek
CZ ID
Neopneumovirus 1
Neopneumovirus 1
Neopneumovirus 2
Neopneumovirus 2
Neopneumovirus 3
Neopneumovirus 3

## Slide 11
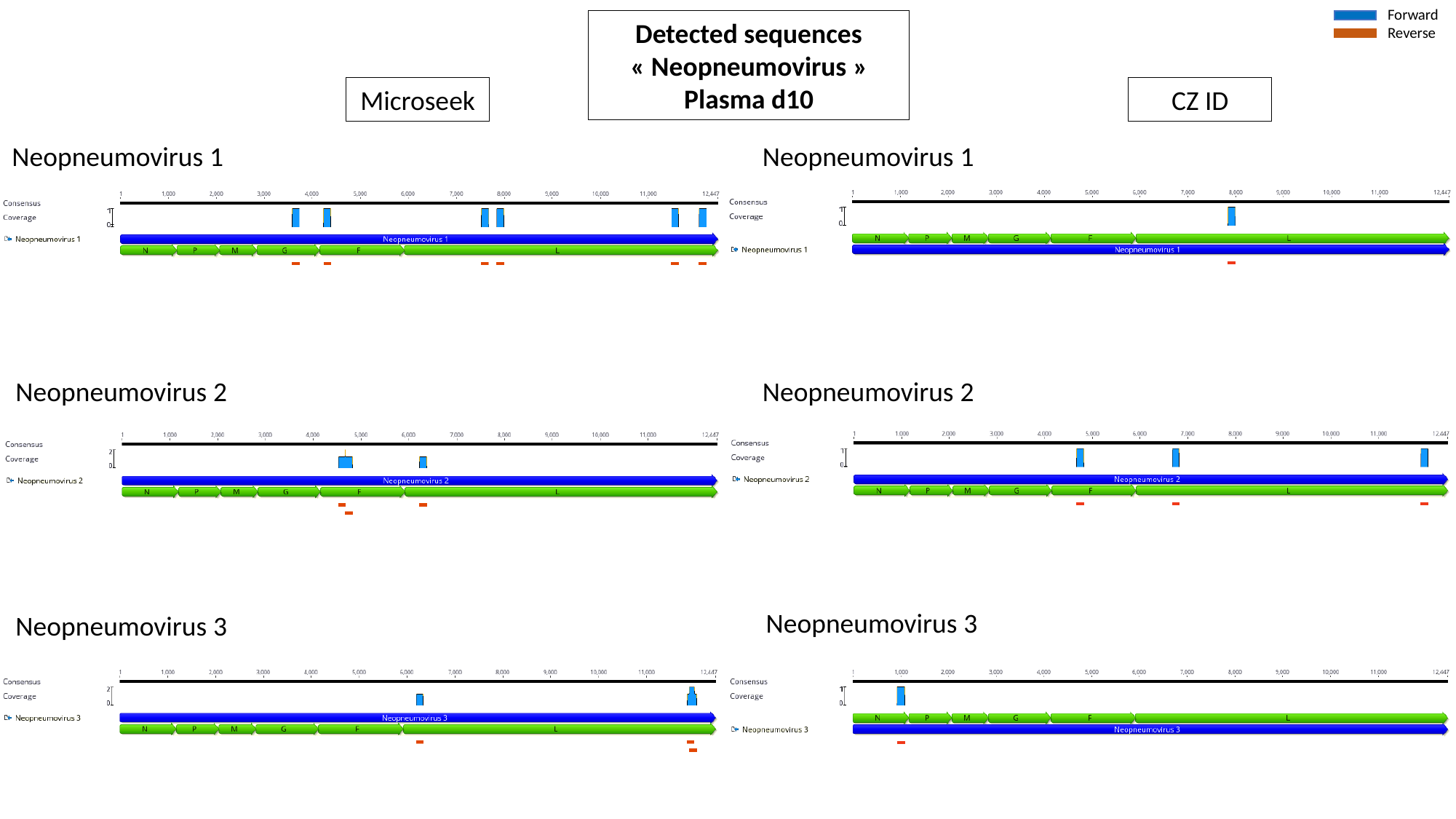

Forward
Reverse
Detected sequences
« Neopneumovirus »
Plasma d10
Microseek
CZ ID
Neopneumovirus 1
Neopneumovirus 1
Neopneumovirus 2
Neopneumovirus 2
Neopneumovirus 3
Neopneumovirus 3

## Slide 12
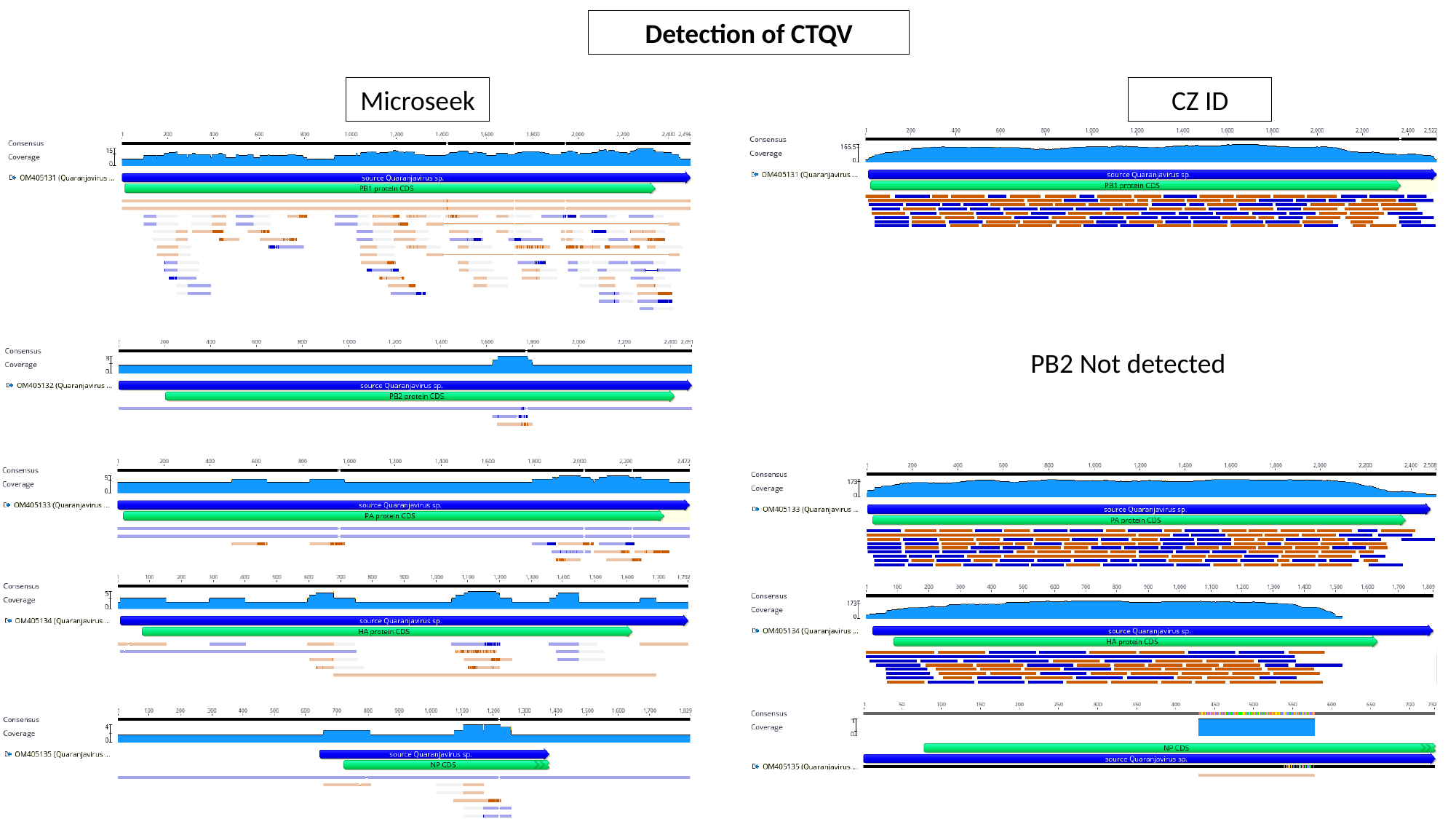

Detection of CTQV
Microseek
CZ ID
PB2 Not detected
