# Supplementary material for: Microseek: A Protein-Based Metagenomic Pipeline for Virus Diagnostic and Discovery
Source: Viruses. 2022 Sep 8;14(9):1990. doi: 10.3390/v14091990 (PMC9500916; doi:10.3390/v14091990)
Supplement: Supplementary file 1 [file viruses-14-01990-s001.zip › TableS1.pdf]

Table S1. Rules followed for the generation of spiked datasets.

**Virus**	**Genome Template**	**Transcripts Template**	**Spiking Rules for the "Tissues" Dataset**
Parvovirus B19 (ssDNA)	DNA genomic strand	DNA genomic strand and reverse complement	Genome template: 10%<br>Transcripts template: 90%; with 90% DNA genomic strand / 10% reverse complement
Epstein–Barr virus (dsDNA)	Both DNA genomic strands	Selection of sense coding mRNA and sense and antisense non-coding RNA	Genome template: 10%<br>Transcripts template: 90%; with 50% sense mRNA (BALF5 & GP350) / 50% sense and antisense non-coding RNA (EBER1 & EBER2)
Coxsackievirus B6 (ssRNA+)	RNA+ genomic strand	RNA+ genomic strand and reverse complement	Genome template: 10%<br>Transcripts template: 90%; with 90% RNA+ genomic strand / 10% reverse complement
Respiratory syncytial virus (ssRNA-)	RNA- genomic strand	RNA- genomic strand and reverse complement	Genome template: 10%<br>Transcripts template: 90%; with 10% RNA- genomic strand / 90% reverse complement
Reovirus 1 (dsRNA)	Both RNA genomic strands	Both RNA genomic strands	Genome template: 10%<br>Transcripts template: 90%; with 50%-50% of both genomic strands
HIV-1 (retrovirus)	RNA+ genomic strand	Sense coding mRNA (gag, pol, env)	Genome template: 10%<br>Transcripts template: 90%; with 100% sense coding transcripts
